# Linkage Mapping vs. Association: A Comparison of Two RADseq‐Based Approaches to Identify Markers for Homomorphic Sex Chromosomes in Large Genomes

**DOI:** 10.1111/1755-0998.70019

**Published:** 2025-07-24

**Authors:** James France, Wiesław Babik, Katarzyna Dudek, Marzena Marszałek, Ben Wielstra

**Affiliations:** ^1^ Institute of Biology Leiden Leiden University Leiden the Netherlands; ^2^ Naturalis Biodiversity Center Leiden the Netherlands; ^3^ Institute of Environmental Sciences Faculty of Biology, Jagiellonian University Kraków Poland

**Keywords:** association‐based approach, linkage map, RADseq, sex chromosomes, sex identification, smooth newt

## Abstract

Reliable tools for the identification of genetic sex are invaluable in many fields of biology, but their design requires knowledge of sex‐linked sequences, which is lacking in many taxa. Restriction‐site‐associated DNA sequencing (RADseq) is widely used to identify sex‐linked markers, but multiple distinct strategies are employed, and it is often not obvious which is most suitable. In this study, we compare two approaches for using RADseq to identify sex‐linked markers. We use the common newt, *Lissotriton vulgaris* , as our study system, providing a challenging combination of homomorphic sex chromosomes and an exceptionally large genome. We attempt an associative approach, sequencing 60 adult newts of known‐sex individuals, and compare this to a linkage mapping approach utilising a family of 146 offspring with unknown sex. After optimisation for a highly paralogous genome, the associative approach identifies five Y‐chromosome‐linked markers in *L. vulgaris* , and we design a robust PCR protocol for molecular sexing of four more related species. Via the linkage approach, we construct a high‐density map featuring 10,763 markers, matching the observed karyotype of *L. vulgaris* and showing broad synteny with the Iberian ribbed newt ( *Pleurodeles waltl* ). However, without incorporating the markers identified via the association‐based approach, we cannot confidently distinguish a sex‐determining region in the linkage map, either by analysing marker density or by identifying clusters of paternal markers. We conclude that linkage mapping alone is unlikely to yield sex‐linked markers in organisms with very small sex‐determining regions, whereas association‐based RADseq can still be effective under these conditions.

## 1 Introduction

Sex‐linked genetic markers are vital to both applied and fundamental biology. In agriculture and aquiculture, molecular markers boost productivity by, for instance, enabling the early selection of fruit‐bearing female date palms (Intha and Chaiprasart 2020) or aiding the maintenance of all‐male stocks of tilapia (Curzon et al. 2021). Molecular sex identification is viable even on small and degraded samples, making it invaluable for ecology, conservation, and forensic biology. Examples include determining the sex of tiger prey from hairs recovered from scat (De et al. 2019), monitoring elephant sex ratios by genotyping dung (Vidya et al. 2003) and identifying the illegal poaching of female pheasants (An et al. 2007). As sex‐determining regions of the genome have been identified as drivers of speciation (Dufresnes and Crochet 2022; Johnson and Lachance 2012; Payseur et al. 2018), their identification and study are of particular importance to evolutionary biology.

Many taxa have highly conserved sex‐determination systems (Cortez et al. 2014; Ellegren 2010), enabling a single method of molecular sex identification to be used across an enormous range of species, with little modification. For example, all birds possess a ZW chromosome system, and a single primer pair based on the CHD1 gene allows for sex identification across the Neognathae, which includes over 99% of bird species (Fridolfsson and Ellegren 1999). Similarly, amplification of the SRY gene identifies the presence of the Y‐chromosome in a wide range of eutherian mammals (Hrovatin and Kunej 2018).

However, such conservation is far from universal, and other branches of the tree of life have experienced frequent turnover of sex chromosomes (Ma and Rovatsos 2022), often made obvious by transitions between male and female heterogamety (Bachtrog et al. 2014; van Doorn and Kirkpatrick 2010). Groups notable for rapid evolution in sex‐determination systems include fish (Kitano et al. 2024), squamate reptiles (Ezaz et al. 2010) and amphibians (Miura 2017). In addition, while the majority of plants are hermaphroditic (or monoecious), dioecy (two fully separate sexes) has independently evolved on numerous occasions (Renner 2014). Rapid turnover complicates molecular sex identification, as new markers may have to be identified on a lineage‐by‐lineage basis. Exacerbating this, evolutionarily young sex chromosomes are typically not highly differentiated, resulting in homomorphic chromosomes with only a small region in which sex‐linked markers may be found (Charlesworth et al. 2005; Wright et al. 2016).

Several sequencing techniques are employed for the identification of sex‐linked markers. Recent studies have tended to employ either whole genome sequencing (WGS) (Darolti et al. 2019; Keinath et al. 2018; Rafati et al. 2020) or restriction‐site‐associated DNA sequencing (RADseq) (Gamble et al. 2015; Hime et al. 2019; Hu et al. 2019). WGS is a powerful technique, but the cost of sequencing the entire genome of multiple individuals of both sexes may be prohibitive. This is particularly the case for organisms with exceptionally large genomes, such as salamanders, lungfish and many genera of dioecious plants such as ginkgo, mistletoe and yew, that all have genome sizes in excess of 10 Gbp (Gregory 2024; Pellicer and Leitch 2020). For such gigantic genomes, RADseq may be a superior technique. RADseq targets restriction‐site‐bounded sequences scattered randomly throughout the genome (Miller et al. 2007), giving genome‐wide data, while requiring orders of magnitude less sequencing than WGS.

There are multiple approaches to designing a study identifying sex‐linked RADseq markers. The most conceptually simple design is association‐based, exploiting pre‐existing knowledge of the sex of samples to associate markers with a particular sex (Gamble and Zarkower 2014). For example, the tool RADsex (Feron et al. 2021) operates by taking RADseq data from individuals of both sexes and screening for markers that are present in one sex and absent in another. The RAD marker set can also be screened for SNPs present only in one sex, markers present at twice the copy number in one sex, and markers that show significant genetic differentiation between sexes (via *F*_st_) (Brelsford et al. 2017; Trenkel et al. 2020). This study design typically requires 10–30 individuals per sex, with decreasing sample sizes increasing the risk of generating false positives. In situations where morphologically sexed samples are difficult to obtain, this requirement may become disadvantageous.

An alternative approach is to leverage the lack of recombination in sex‐linked regions. This offers the advantage of not requiring the sex of the samples to be known in advance, allowing the use of juvenile specimens, or even embryos, if enough DNA can be obtained. For example, the recently published method SLRfinder (Yi et al. 2024) first maps RADseq (or WGS) reads to a reference genome assembly before identifying clusters of SNPs showing linkage disequilibrium as candidates for a sex‐linked region. For taxa without an available genome assembly, a linkage map may be constructed instead. As RADseq involves many thousands of markers scattered randomly across the genome, it is ideal for building high‐density linkage maps. Sex‐linked regions and the associated makers can then be detected by identifying clusters of SNPs unique to the heterogametic parent (Hu et al. 2021) or by locating regions of reduced recombination (Brelsford et al. 2016). A linkage map requires more investment than an association‐based study design, as a linkage family (or families) must be bred and typically at least 100 individuals must be sequenced. However, as the sex of the offspring does not have to be known (Brelsford et al. 2016), a linkage mapping approach may be more feasible in cases where large numbers of adults are not available to be morphologically sexed and juveniles are readily bred but difficult to sex. Additionally, the linkage map itself may be a valuable resource used for purposes such as quantitative trait locus analysis (Peng et al. 2016), exploring the genomic landscape of recombination (Apuli et al. 2020) or anchoring scaffolds of a whole genome assembly (B.‐Y. Lee et al. 2019).

For identifying sex‐linked markers in organisms with large genomes and no available genome assembly, both association‐based and linkage mapping RADseq study designs appear to offer advantages. Importantly, these approaches require significantly different sample sets, and as new samples often cannot be acquired at short notice, it is pertinent to choose the most appropriate study design ahead of time. However, it is not always clear which methodology is more likely to yield useful sex‐linked markers. Most studies employ a single methodology, and while this does provide a list of strategies that have proved successful in at least one situation, the publication bias against negative results means that the limitations of these approaches remain obscure. The publications that do compare different tools for sex‐linked marker discovery tend to either be reviews, which aggregate results generated in wildly different contexts (Palmer et al. 2019), or comparisons of different statistical approaches for the identification of sex‐linked regions (Trenkel et al. 2020). In this study, we aim to contrast two approaches to sex‐linked marker discovery using RADseq (linkage mapping and association with known morphological sex) by applying both to a single, challenging species, the common newt ( *Lissotriton vulgaris* ).

*Lissotriton vulgaris* is one of the most widely distributed amphibian species in Europe, ranging from Ireland to Siberia (Sparreboom 2014). It is part of the smooth newt species complex, which includes six closely related newt species found in Europe and western Asia (Pabijan et al. 2017; Wielstra et al. 2018). Beyond the smooth newt complex, the genus *Lissotriton* includes four additional species, more distantly related to *L. vulgaris* (Babik et al. 2005; Mars et al. 2025). Like all salamanders, *Lissotriton* has gigantic genomes, estimated at 27.7–32.0 Gbp (Gregory 2024; Litvinchuk et al. 2007). *Lissotriton* possess XY sex‐determination systems with little to no heteromorphism (Schmid et al. 1979; Zboźeń and Rafiński 1993). No Y‐linked marker has previously been reported in any salamander; however, RADseq studies have identified the ancestral amphibian ZW system in the family Cryptobranchidae (Hime et al. 2019; Hu et al. 2019, 2021), and the first salamander whole genome assembly revealed a tiny 300 Kbp W‐linked region in the axolotl (Keinath et al. 2018).

We first attempt to identify Y‐linked markers via the associative approach by performing RADseq on a group of known‐sex *L. vulgaris* and identifying sex‐linked presence/absence markers. We then test the linkage mapping approach to identify a Y‐linked region, gathering RADseq data from a full‐sibling *L. vulgaris* family with offspring of unknown sex. Finally, we validate candidate markers by PCR amplification in multiple taxa within the genus *Lissotriton*.

## 2 Methods

### 2.1 Sample Acquisition

For identification of Y‐linked markers by association, samples from 60 sexed‐adult *L. vulgaris* (30 male, 30 female) were collected from the Kraków metropolitan area in Poland; these samples are also reported in Babik et al. (2024). For the construction of the linkage map, an *L. vulgaris* family was bred, consisting of two parents (one adult male and one adult female, collected in Kraków, Poland) and 146 offspring of unknown sex. For screening of candidate markers via PCR, we employed pairs of known‐sex samples (one male and one female per taxon) for *L. vulgaris* and multiple taxa belonging to the smooth newt complex (*L. v. ampelensis*, *L. v. meridionalis*, *L. montandoni* , *L. graecus*, *L. kosswigi* and *L. schmidtleri* ), as well as more distantly related *Lissotriton* species ( *L. boscai* , *L. helveticus* and *L. italicus* ). For validation of the diagnostic PCR protocols, we employed an additional 41 *L. vulgaris* (22 males and 19 females) and 44 *L. montandoni* (23 males and 21 females). Samples were obtained from localities across Europe and Anatolia (Table S4). Samples from adults consisted of tail tips, and for offspring, the freshly hatched larvae were collected whole. After collection, samples were stored in 96% ethanol.

### 2.2 DNA Extraction, Library Preparation and RAD‐Sequencing

Whole genomic DNA was extracted from the selected tissue samples with the Promega Wizard Genomic DNA Purification Kit (Promega, Madison, WI, USA), according to the salt‐based extraction protocol of Sambrook and Russell (2001). Double‐digest RADseq libraries were prepared according to the Adapterama III High‐Throughput 3RAD protocol (Bayona‐Vásquez et al. 2019) from 100 ng of genomic DNA, using restriction enzymes *Eco*RI, *Xba*I, and *Nhe*I. Fragments in the range of 490–600 bp were excised using Pippin Prep, the libraries were pooled equimolarly and 150 bp paired‐end sequencing was performed by Novogene (Cambridge, UK) on the Illumina NovaSeq 6000 (Illumina Inc., San Diego, CA, USA) platform, targeting a yield of 1 Gbp per sample for the linkage map family and 2 Gbp for the known‐sex adults.

### 2.3 RADseq Data Processing With Stacks

The Stacks package v2.54 (Catchen et al. 2013; Rochette et al. 2019) was employed to process raw reads from all RADseq samples. Reads were demultiplexed and trimmed via the *process_radtags* program. The *denovo_map.pl* pipeline was then used to group reads into putative loci. Default settings were used except for the parameter *M*, which controls how many mismatched bases two read‐pairs may have and still be assigned to the same locus. If M is too low, Stacks may not correctly group reads from the same locus together (especially if samples are genetically divergent); however, if *M* is too high it may result in reads from paralogous loci being inappropriately aggregated together (Paris et al. 2017). As our RADseq analysis was based on two sample sets, which would not be expected to exhibit great genetic diversity (a captive‐bred linkage map family and a group of wild‐caught newts from a relatively small area), a low value of *M* would seem appropriate. Accordingly, we selected the default value of *M* = 2 for the linkage family, as this low value will minimise the distortions caused by mapping paralogs together as falsely heterozygous loci.

However, we hypothesised that an overly high value of M may actually be helpful when selecting markers for molecular sex identification. This increases the chance that sex‐linked loci with autosomal paralogs will be assigned reads even in the opposite sex, and so filtered out in subsequent analysis. This is desirable as these loci would likely give false positives in PCR‐based genotyping (due to amplification of the paralog). Consequently, we ran our analysis of the known‐sex RADseq data three times, with values of *M* = 2, *M* = 6, and *M* = 10.

### 2.4 Sex‐Associated Presence/Absence Marker Discovery

The bam files produced from each of the three runs of *denovo_map.pl* were processed with the *depth* function of SAMtools (Danecek et al. 2021) to produce a table of the number of reads of each marker in each of the sexed‐adult samples. A custom R script was then used to identify candidate Y‐linked markers which had reads in at least 90% of male samples (with a median depth of at least 20) and < 10% of female samples.

We aimed to minimise the likelihood of candidate Y‐linked markers failing in PCR validation by avoiding candidates with a large number of paralogous sequences present in the genome. Primers designed for such markers would have a high chance of amplifying products from autosomal paralogs, resulting in false positives in female samples. Therefore, a BLAST (Camacho et al. 2009) search was then conducted for each candidate against the catalogue of all RAD markers found in the run of the same M value, and the number of potential paralogous hits (> 80% sequence similarity with a query coverage of > 25%) recorded. Candidate markers were ranked based on the absence of residual reads in females, the number of potential paralogs, and average read depth in males. The ten highest‐ranked candidate markers from each run were selected for PCR screening.

After removing any duplicate markers (where the same sequence was selected from multiple runs of the pipeline), primers were designed with Primer 3 (Untergasser et al. 2012) targeting an optimal primer length of 20 bp and melting temperature of 60°C. To facilitate the design of a multiplex PCR, for each marker we attempted to design two primer pairs, amplifying both a shorter (ca. 100 bp) and a longer (ca. 200 bp) product. For candidate markers that did not consist of a continuous sequence, as the RAD fragments were longer than 2 × 150 reads, the primer pairs amplifying the shorter products were derived entirely from the forward read, whereas the longer product would incorporate an additional sequence of unknown length between the forward and reverse reads. Sequences of all primers are found in Table S3.

To compare different bioinformatic approaches to identify sex‐associated presence/absence markers, we also used the RADsex package (Feron et al. 2021), which is designed specifically for this process. Unlike Stacks, RADsex does not natively support paired‐end reads; however, it is still possible to use the first read of each pair as an input. Additionally, RADsex does not attempt to assign slightly mismatched reads to the same locus, so it acts similarly to Stacks set to *M* = 0. Starting with the demultiplexed reads produced by the *process_radtags* program of Stacks, we used the RADsex commands *process* and *distrib* with all parameters set to their default values. We then filtered the resulting markers for a median depth of at least 20 in male samples to produce a list of candidate markers. A BLAST (blastn) (Camacho et al. 2009) search (> 80% sequence similarity with a query coverage of > 25%) for each candidate was then conducted against the catalogue of all RAD markers identified by RADsex to identify paralogous markers. Due to the short sequence length and high expected number of paralogs, we did not design primers for the markers identified in RADsex; however, we incorporated them into the linkage map in the same manner as with the markers identified via Stacks.

### 2.5 Sex‐Associated Marker Validation

The primers designed for candidate sex‐associated markers were tested via PCR amplification with 2× QIAGEN multiplex master mix (QIAGEN B.V, Venlo, Netherlands). After optimisation, a final PCR protocol was designed consisting of a 95°C hot start for 10 min, followed by 35 cycles of denaturation for 30 s at 95°C, 60 s annealing at 61°C and 45 s extension at 72°C, with a final extension of 10 min at 72°C. All primers were used at a final concentration of 0.1 μM. Initial screening was against a single male/female pair of *L. vulgaris* . Markers showing male‐specificity were validated against a panel of six male and six female *L. vulgaris* . Validated markers were then tested against a male/female pairs of both *L. montandoni* and *L. helveticus* (as representatives of the *L. vulgaris* complex, and the wider *Lissotriton* genus, respectively). Any markers with multispecies sex‐specificity were tested in male/female pairs of all available species of *Lissotriton*. Finally, a multiplex PCR was designed, combining the most broadly male‐specific markers with an autosomal control marker, CDK‐17 (Meilink et al. 2025), which amplifies a product of 537 bp. This multiplex PCR was then validated in the male/female pairs of all species. Additional validation was performed using the original 60 known‐sex *L. vulgaris* adults used for RADseq, and an additional panel consisting of 45 *L. vulgaris* (24 males and 21 females, including the 12 individuals from the panel described above, and the parents of the linkage map) and 47 *L. montandoni* (24 males and 22 females). As *L. montandoni* is the most divergent lineage within the *L. vulgaris* species complex (Mars et al. 2025), these large sample sets form a useful phylogenetic bracket.

### 2.6 Linkage Map Construction

The joint VCF file produced by Stacks was filtered with VCFtools (Danecek et al. 2011) to exclude indels and SNPs with > 5% missing data, a mean depth of < 10, or a minor allele frequency of < 0.2 (note that, as the expected minor allele frequency of Y‐linked markers would be 0.25, this strict filtering should be relaxed if this methodology is adapted to data sets with fewer samples). The *thin* function of VCFtools was used to select a single SNP per marker. Lep‐MAP 3 (Rastas 2017) was then used to construct paternal, maternal, and sex‐averaged linkage maps.

Because the candidate Y‐linked markers identified in the known‐sex adults are expected to be entirely absent in females, they would appear as missing data within the VCF file. In males, they would appear to be entirely homozygous, due to all reads coming from the single Y‐chromosome. As a result, these markers would be filtered out in several steps of the linkage map construction pipeline and could not be used to calculate linkage in any case (as that would require heterozygosity in at least some samples). To resolve this issue and incorporate the candidate Y‐linked markers into the linkage map, a custom R script was used to translate the presence/absence of reads for these markers into pseudo‐SNP genotype calls for all samples. Markers with no reads were assigned an artificial AA genotype, whereas markers with reads were assigned as AT (this may be thought of as making the ‘absence’ allele on the X chromosome visible to the software). These calls were appended to the call file produced by the first stage of the Lep‐MAP 3 pipeline (the *ParentCall2* module) and incorporated into all subsequent steps.

Initial linkage groups were created with the *SeparateChromosomes2* module, with a LOD limit of 20 and distortion LOD set to 1. Unplaced markers were then added with the *JoinSingles2All* module with a LOD limit of 15. The markers were then ordered with the *OrderMarkers2* module, using 20 merge iterations, 8 polish iterations, a minError value of 0.02 and the scale setting M/N 2. The informative mask options 23 and 13 were used for the paternal and maternal maps respectively –these settings cause Lep‐MAP 3 to ignore markers which are only heterozygous in a particular parent (either the mother or father, depending on the option chosen); hence in the maternal map the Y‐linked markers will not be placed as they are only informative in the father.

In this study, we were primarily concerned with the viability of using linkage to identify sex‐linked regions in scenarios where the offspring sex is unknown. However, if offspring sex were known in advance, this information could be processed by the *ParentCall2* module of Lep‐MAP 3 to identify Z or X‐linked markers. To examine this functionality, we used a custom R script to call the sex of the linkage map offspring based on the presence or absence of the Y‐linked markers identified in the known‐sex adults in the offspring RADseq data. We then used the resulting PED file as an input into *ParentCall2* with the option Xlimit = 2 specified and highlighted any resultant candidate Y/X‐linked markers.

### 2.7 Linkage Map Comparison With *Pleurodeles waltl* Genome

Initial validation of the linkage map was performed by BLASTing the sequences of the mapped markers against the genome assembly of the Iberian ribbed newt ( *Pleurodeles waltl* ) (Brown et al. 2025), using a word size of 11 and requiring a minimum E value of 1e−20. To account for the high degree of paralogy typical of newt genomes, the BLAST results were filtered to include only hits that exceeded the significance of the next best hit by five orders of magnitude, following the methodology of Purcell et al. (2014). The filtered BLAST hits were then used to create an Oxford plot via a custom R script.

### 2.8 Marker Density Analysis

In an XY system, the sex‐linked region is typically not expected to undergo recombination in males (although in evolutionary young sex‐determination systems, this non‐recombining region may be very small), and so markers within this region should exhibit extreme genetic linkage when transmitted from father to offspring. When a paternal linkage map is constructed, these markers will form a region of high marker density. However, as the sex‐linked region does undergo recombination in females, these markers should be spread over a wider region when the corresponding maternal map is constructed. We attempted to locate the sex‐linked region by plotting marker density in both paternal and maternal linkage maps via a custom R script and identifying a peak which is exclusive to the paternal map.

### 2.9 Analysis of Paternal‐Specific Markers and SNPs

To be placed on the linkage map, a marker must have an SNP that is heterozygous in at least one of the parents. SNPs which are heterozygous in the father but homozygous in the mother are termed paternal‐specific SNPs. RAD loci which include only paternal‐specific SNPs are termed paternal‐specific markers. While a large number of paternal (and maternal) specific SNPs and markers will be randomly scattered across the genome, the Y‐linked region is expected to be particularly enriched in paternal‐specific SNPs and markers, as in an XY system, this region is, by definition, heterozygous in males. To identify this enriched region, we first plotted the number of paternal SNPs and markers against the total number of markers per linkage group. We then used a custom R script to divide each linkage group into bins of 2 cM and plot the probability (assuming the markers were randomly and independently distributed) of obtaining the measured number of paternal‐specific markers and SNPs, using a binomial distribution for the markers and a Poisson distribution for the SNPs.

## 3 Results

### 3.1 Sex Association in Known‐Sex Adults

After demultiplexing and filtering, the 60 known‐sex individuals yielded a total of 901 million reads (per sample median: 13.7 M, interquartile range: 11.5–16.5 M). The three runs of the denovo_map.pl pipeline yielded differing results as parameter M was varied. Increasing M decreased the total number of loci identified in the dataset (*M* = 2: 1,541,940, *M* = 6: 1,026,619, *M* = 10: 911,354) and increased the mean adjusted sequencing depth per sample (*M* = 2: 28.2, *M* = 6: 30.6, *M* = 10: 31.7) and the proportion of loci found in at least 50% of samples (*M* = 2: 8.61%, *M* = 6: 12.56%, *M* = 10: 13.31%).

As expected, in the initial sets of candidate markers (*M* = 2: 32, *M* = 6: 26, *M* = 10: 19, after duplicates removed, 35 unique sequences in total), selected by screening for presence in males and absence in females, a high degree of paralogy was observed, with the number of BLAST hits per marker varying from 1 to 500. The proportion of candidate markers without paralogous hits increased with parameter M (*M* = 2: 19%, *M* = 6: 38%, *M* = 10: 53%).

After the 10 highest‐ranked candidate markers from each run were selected, the majority of candidates appeared in multiple runs. Removing duplicates left 14 unique candidate markers for PCR validation. In total, 25 primer pairs were designed (Table S2), as for three markers, Primer3 failed to generate a valid primer pair for the ‘short’ product under the given conditions.

Via RADsex a total of 24 candidate Y‐linked markers were identified, 14 of which were identical to markers obtained above and 10 of which were new. As expected, the number of paralogs in this group of markers was significantly higher, with all markers having at least 50 paralogous BLAST hits.

### 3.2 Sex‐Associated Marker Validation

**FIGURE 1:**
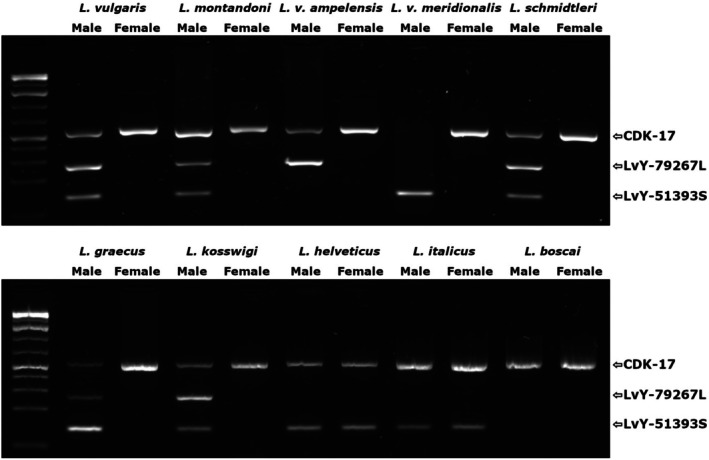
The results of the multiplex PCR designed for molecular sex identification in the *L. vulgaris* species complex. The three primer sets amplify a control marker CDK‐17 (537 bp) and two Y‐linked markers LvY‐79,267‐Long (ca. 240 bp) and LvY‐51,393‐Short (124 bp). Within the *L. vulgaris* complex ( *L. vulgaris* , *L. montandoni* , *L. schmidtleri* , *L. graecus* and *L. kosswigi* ) amplification of the Y‐linked markers is observed only in male samples. In more distantly related *Lissotriton* species ( *L. helveticus* , *L. italicus* and *L. boscai* ) sex‐specific amplification is not observed. As male amplification of one of the Y‐linked markers is not observed in each of the non‐nominate subspecies of *L. vulgaris* (LvY‐51,393‐Short in *L. v. ampelensis* and LvY‐79,267‐Long in *L. v. meridionalis*) we recommend using both diagnostic primer pairs for reliable sex identification.

Of the 14 candidate markers, six show validated male‐specific amplification in *L. vulgaris* , with an additional marker showing initial male‐specificity screening but failing in the 12 individual validation panels. Three markers retain sex‐specificity in *L. montandoni* (Table 1). Two markers, LvY‐79,267 and LvY‐51,393, show broad male‐specificity within the smooth newt species complex. However, both markers fail to amplify in males in at least one taxon within this group, with LvY‐79,267 failing in *L. v. meridionalis* and LvY‐51,393 failing in *L. v. ampelensis*. In the more distantly related species ( *L. helveticus* , *L. italicus* and *L. boscai* ) no sex‐specific amplification is observed with any primer pair, with markers failing to amplify in either sex or amplifying in both sexes (all gels resulting from screening and validation found in Figure S1). If the candidate markers from each run are considered separately, higher values of parameter *M* give more useful results. Five out of ten candidates selected from *M* = 10 are sex‐specific in *L. vulgaris* , compared to four out of ten from *M* = 6 and two out of ten selected from *M* = 2. A final multiplex mix (Table 2), amplifying fragments from both LvY‐79,267 and LvY‐51,393, as well as the autosomal control marker CDK‐17, shows robust sex identification across all taxa within the *L. vulgaris* species complex included in this study (Figure 1). We also observe that this multiplex mix correctly identifies the sex of all 105 *L. vulgaris* and 47 *L. montandoni* samples included in the larger validation sets (Figures S5 and S6).

**TABLE 1:** Summary of results of PCR screening of primer pairs designed for candidate Y‐linked markers in *Lissotriton* newts.

Marker	Ranked in Stacks run *M*=			Primer pair	Male‐specific amplification in *Lissotriton* taxa									
					*L. vulgaris* species complex							Other		
	2	6	10		*L. vulgaris*	*L. montandoni*	*L. v. ampelensis*	*L. v. meridionalis*	*L. schmidtleri*	*L. graecus*	*L. kosswigi*	*L. helveticus*	*L. italicus*	*L. boscai*
lvY‐36,220	Yes	Yes	Yes	Short	○									
				Long	×									
lvY‐65,590	Yes	Yes	Yes	Short	○									
				Long	○									
lvY‐81,842	Yes	Yes	Yes	Short	○†	○						×		
				Long	○†	×						×		
lvY‐123,701	Yes	Yes	Yes	Short	+	×						×		
				Long	+	×						×		
lvY‐138,925	Yes	Yes	Yes	Short	○									
				Long	○									
lvY‐143,365	Yes	Yes	Yes	Short	○									
				Long	○									
lvY‐79,267		Yes	Yes	Short	+	+	+	×	+	+	+	×	×	×‡
				Long	+	+	+	×	+	+	+	×	×	×‡
lvY‐115,632		Yes	Yes	Short	+	+	×	×	○	+	+	×	○	×
				Long	+	+	○	○	○	○	○	×	×	×‡
lvY‐128,014		Yes	Yes	Short	+	×‡						×		
				Long	○									
lvY‐51,393	Yes		Yes	Short	+	+	×	+	+	+	+	○	○	×‡
				Long	+	×	×	+	×	+	+	○	○	×‡
lvY‐99,941		Yes		Long	○									
lvY‐11,521	Yes			Long	○									
lvY‐28,978	Yes			Long	○									
lvY‐102,891	Yes			Short	○									
				Long	○									

*Note:* Results are indicated as: + amplification only in male samples, ○ amplification in both male and female samples, × no amplification in either sex. Twenty‐five primer pairs were tested in a male–female pair of *L. vulgaris* , followed by validation of successful markers in a 12 individual panel. Nine primer pairs, covering five candidate markers, show confirmed male‐specific amplification in *L. vulgaris* , with a further two primer pairs, marked with †, showing initial male‐specificity but failing in the wider panel. The successful primer sets were then tested in male–female pairs of *L. montandoni* and *L. helveticus* , and the three markers which show sex‐specificity in *L. montandoni* were then tested in male–female pairs of all available *Lissotriton* taxa. While markers lvY‐79,267 and lvY‐51,393 demonstrate broad male‐specificity within the *L. vulgaris* species complex, no marker shows any sex‐specificity in more distantly related *Lissotriton* taxa. In several cases, marked with ‡, the PCR results are difficult to interpret due to faint amplification of multiple off‐target bands.

**TABLE 2:** Primer sequences used for the sex diagnostic multiplex PCR for use within the *L. vulgaris* species complex, as demonstrated in Figure 1.

Primer pair	Forward sequence	Reverse sequence	Product length (bp)
CDK‐17	GGCATGGGAAGAACAGAAGA	CCATCTGCTTGGACTGTTGA	537
lvY‐51,393‐short	GACCACTGTAGAGGAGGTTGG	GCTGCCTGTTTCTGGATGTC	124
lvY‐79,267‐long	CAAGGCCAAAATGATCCCGC	TGTGCATTGACCATAAAGCCC	ca. 240

*Note:* CDK‐17 is a control marker which amplifies in all species, lvY‐51,393‐short and lvY‐79,267‐long amplify only in males, however inclusion of both is recommended as some taxa may fail to amplify one the markers.

### 3.3 Linkage Map Construction

The 148 individuals of the linkage map family yielded a total of 1.34 billion reads (per sample median: 7.91 M, interquartile range: 7.04–8.92 M). The denovo_map.pl pipeline produced a total of 414,146 RAD loci, of which 137,538 (33.2%) were present in at least 50% of individuals. After filtering with VCFtools, a total of 16,738 markers (each with 1 representative SNP) were available for linkage map construction.

**FIGURE 2:**
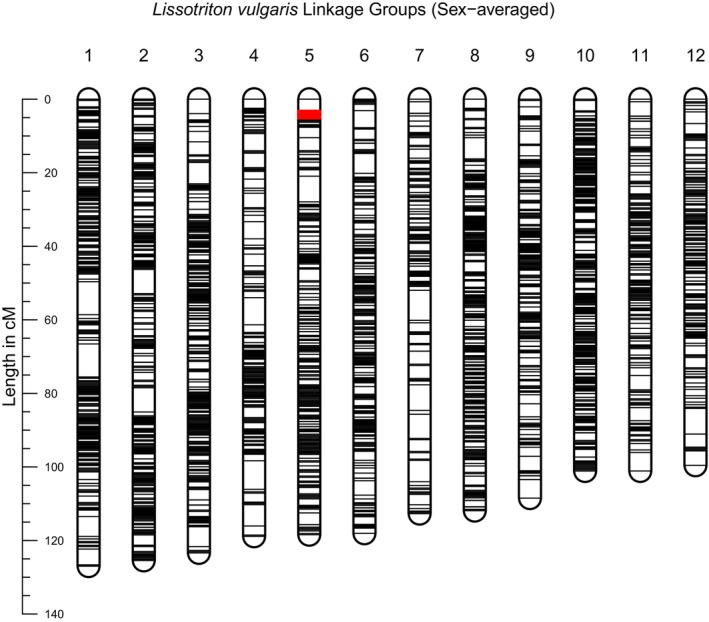
Sex‐averaged linkage map for *L. vulgaris* based on a full‐sib family of 146 offspring. The linkage map is composed of 10,763 RAD markers in 12 linkage groups, ordered by length in centimorgans. Thirty‐two Y‐linked presence/absence markers (highlighted in red), first identified in known‐sex adult *L. vulgaris* are located within a 2 cM region of Group 5, identifying this as the Y‐chromosome.

The final linkage maps consist of 12 linkage groups. The sex‐averaged map contains 10,763 markers and has a total length of 1366 cM (Figure 2). Respectively, the paternal and maternal maps (Figures S7 and S8) contain 7484 and 7452 markers and have total lengths of 1300 and 1688 cM (a more detailed overview is found in Table S1). The sex‐averaged and paternal maps include 32 Y‐linked presence/absence markers. In both maps, these form a tight cluster, spanning < 2 cM, located at one end of linkage group 5. If the male‐associated markers identified via RADsex are incorporated into the linkage map (Figure S9), they also localise entirely within this cluster. If the input of *ParentCall2* is augmented with information of the sex of the offspring, an additional eight Y/X‐linked markers are identified, of which seven cluster within 2 cM of the presence/absence markers (Figure S10).

### 3.4 Comparison With *Pleurodeles waltl* Genome

**FIGURE 3:**
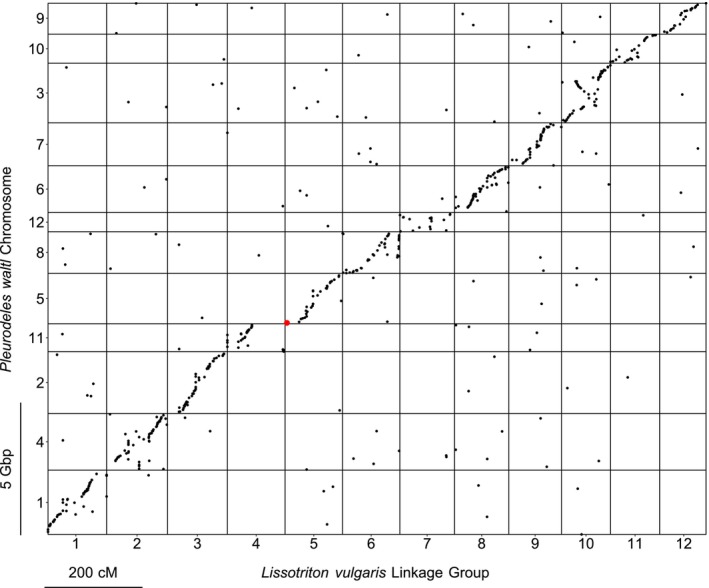
Oxford plot comparing the locations of 579 RAD markers in the *L. vulgaris* linkage map with their orthologs within the *P. waltl* genome, as assembled by (Brown et al. 2025). Two Y‐linked presence/absence markers are highlighted in red. Four hundred and seventy‐two markers (82%) map to orthologous chromosomes, demonstrating broad synteny between the two newt genera.

Five hundred and seventy‐nine (5.4%) of the markers placed on the linkage map, including two Y‐linked markers, can be aligned with sequences within the *P. waltl* genome assembly (Figure 3, see Table S2 for an overview of the distribution of the RAD markers within the *P. waltl* genome). Synteny between the taxa appears strongly conserved, with each linkage group reciprocally matching a single *P. waltl* chromosome, 472 (82%) *L. vulgaris* markers mapping to their orthologous chromosome, and large blocks of conserved synteny are observed within each chromosome/linkage group pair. Evidence of a large inversion is also seen on linkage group 10. We observe no clear pattern in the 18% of markers mapping to non‐orthologous chromosomes, indicating that these are likely a result of misalignment of paralogous sequences, rather than evidence of any large‐scale genomic rearrangements. Linkage group 5 is clearly orthologous to *P. waltl* chromosome 5. The two Y‐linked presence/absence markers that have identifiable orthologs both align with sequences close to one end of chromosome 5, with start co‐ordinates of 35.2 and 62.5 Mbp (the overall length of chromosome 5 is 1.91 Gbp).

### 3.5 Marker Density Analysis

**FIGURE 4:**
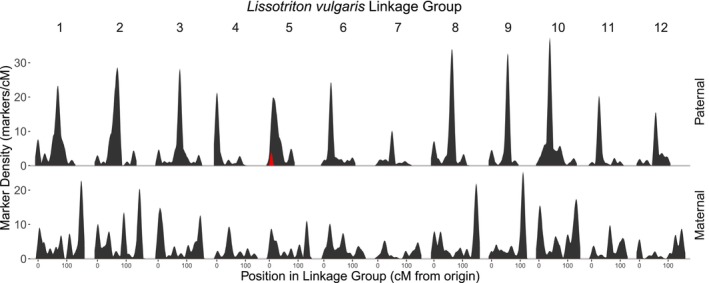
Marker density in the paternal and maternal *L. vulgaris* linkage maps. Total maker density is shown in black, and density of Y‐linked presence/absence markers is shown in red, highlighting the Y‐linked region on linkage group 5. Markers were aggregated into 2 cM bins and a Gaussian smoothing function with a 10 cM range was applied.

Marker density differs significantly between paternal and maternal linkage maps (Figure 4). In the male map, each linkage group is dominated by a single, tight cluster of markers, indicating large regions of reduced recombination. The clusters are usually in the centre of the group, suggesting that most recombination events occur near the ends of the chromosomes. In the maternal map, marker density is more uniform, although areas of increased marker density are observed towards the end of some linkage groups. As expected, the Y‐linked presence/absence markers are found in a region that shows high marker density in the paternal map but not in the maternal map. However, such regions can be observed across the linkage map, which is an expected consequence of the differing rates of recombination in male and female meiosis. While linkage group 5 does show the greatest difference in length and average marker density between the paternal and maternal maps of any of the groups, the peak of marker density in six other paternal linkage groups exceeds that of the Y‐linked region. Peak marker density in the paternal map is found in linkage group 10, where 337 markers map to a single point.

### 3.6 Paternal‐Specific Markers and SNPs

**FIGURE 5:**
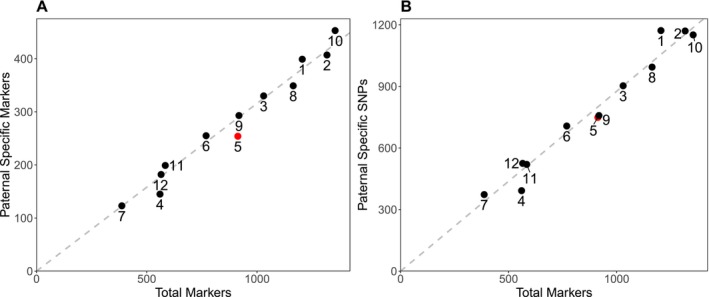
Plots showing the number of the paternal‐specific markers (A) and SNPs (B) against the total number of markers in each group of the sex‐averaged *L. vulgaris* linkage map. Linkage group 5, baring the Y‐linked presence/absence markers is highlighted in red. The distribution of paternal markers and SNPs follows a linear trend.

A total of 3389 paternal‐specific markers and 9414 paternal‐specific SNPs were located within the sex‐averaged linkage map. Distribution across linkage groups was extremely uniform, with a linear trend observed between the number of total markers and paternal‐specific markers/SNPs within each linkage group (Figure 5). Neither linkage group 5 nor any other group deviated significantly from this trend.

**FIGURE 6:**
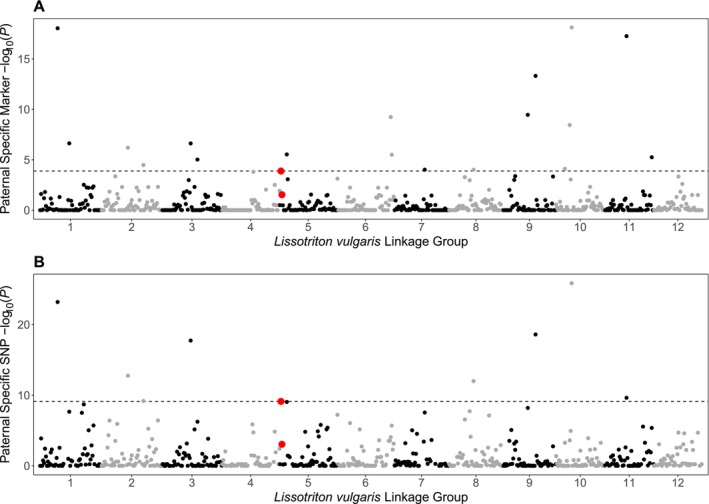
Manhattan style plot showing enrichment in paternal‐specific markers (A) and SNPs (B) in the sex‐averaged *L. vulgaris* linkage map, divided into 689 bins of length 2 cM. Enrichment is shown as ‐log_10_ of the probability of a bin containing the observed number of paternal‐specific markers and SNPs, assuming that all markers have an equal an independent chance of containing paternal‐specific SNPs. The 32 Y‐linked presence/absence markers are located in two adjacent bins highlighted in red. As expected, these bins show significant enrichment in paternal‐specific markers (*p* = 1.38 × 10^−4^) and SNPs (*p* = 7.77 × 10^−10^). However other regions of the genome show even greater enrichment, with 18 and 8 bins exceeding the significance of the Y‐linked region (shown as the dashed horizontal line) in paternal‐specific markers and SNPs respectively.

The region containing the majority of the Y‐linked presence/absence markers has a significantly elevated concentration of both paternal‐specific markers (*p* = 1.38 × 10^−4^) and SNPs (*p* = 7.77 × 10^−10^); however, more significant concentrations are present at multiple locations throughout the linkage map (Figure 6). Eighteen 2 cM bins have a more significant concentration of paternal‐specific markers, and eight have a more significant concentration of paternal SNPs.

## 4 Discussion

We successfully identified sex‐linked presence/absence markers in the smooth newt, *L. vulgaris* via an associative RADseq approach, confirming that this method remains effective even in exceptionally large genomes, such as those of salamanders. As such, genomic gigantism is often the result of the accumulation of repetitive elements (Lee and Kim 2014; Sun et al. 2012), there is a high chance that any given sequence will have multiple paralogs throughout the genome. Our results indicate that the efficiency of the discovery process can be significantly enhanced by aggressively filtering out candidate markers showing such paralogy. Out of 14 markers we test via PCR, five show validated sex association in *L. vulgaris* . Compared to the two previous studies using a similar methodology in salamanders, this is a notably high success rate. Hime et al. (2019) screened 43 loci for sex association in the hellbender, *Cryptobranchus alleganiensis* , with four successful in PCR. Hu et al. (2019) designed 100 candidate primer pairs to yield four reliable W‐linked markers in the Chinese giant salamander, *Andrias davidianus* . In addition, our experience indicates that thoughtful optimisation of the upstream bioinformatics increases the chance of identifying a useful marker. Increasing the value parameter M in the *denovo_map.pl*/*ustacks* programs of the Stacks package from 2 to 10 vastly reduced the chance of a marker with paralogs being selected as a sex‐linked candidate and doubled the number of candidates that gave sex‐specific PCR amplification.

To our knowledge, these presence/absence markers are the first tool for genetic sex identification described in any species of newt (subfamily Pleurodelinae) or salamander within the family Salamandridae. Two markers, LvY‐79,267 and LvY‐51,393, are particularly notable, as we show that in combination they allow for molecular sex identification across the smooth newt species complex with a simple multiplex PCR protocol. As *Lissotriton* take 2–3 years to reach sexual maturity, and are difficult to morphologically sex as juveniles (Sparreboom 2014), a robust genetic assay of sex will be of significant benefit for researchers interested in the conservation, ecology and behaviour of these species.

The sex‐specificity of the markers decreases with phylogenetic distance. Five markers are male‐specific in *L. vulgaris* . Three of these retain specificity in *L. montandoni* –the most divergent lineage within the smooth newt complex (Mars et al. 2025), which is notable as previous cytological studies were unable to identify any sex chromosome in this species (Zboźeń and Rafiński 1993). While we were unable to obtain known‐sex samples of the Caucasian smooth newt *L. lantzi* , it is a member of the smooth newt complex (Wielstra et al. 2018) and so we predict that the identified markers will also be sex‐specific in this species. No marker is found to amplify sex‐specifically in more distantly related *Lissotriton* species. Variation in the Y‐chromosome may contribute to the differing degrees of reproductive isolation within the genus (Johnson and Lachance 2012; Yoshida et al. 2014) – while species within the smooth newt complex hybridise readily, *L. vulgaris* and *L. helveticus* (the palmate newt) show almost complete reproductive isolation, despite co‐occurring over a large area of western Europe (Miralles et al. 2024).

We construct a high‐density linkage map for *L. vulgaris* and identify a Y‐determining region at the end of linkage group 5 via the incorporation of the markers identified above. The resulting map matches the observed karyotype of *L. vulgaris* (Wickbom 1945), and the number and density of markers are significantly increased compared to *L. vulgaris* × *L. montandoni* linkage maps published by Niedzicka et al. (2017). A disadvantage of RADseq‐based linkage maps is that the information they provide typically lacks context; the mapped sequences are unlikely to have any known function, and it is difficult to directly relate a given linkage group to a particular chromosome. This is indeed the case in *L. vulgaris* , where, apart from the acrocentric chromosome 7, all chromosomes are metacentric or submetacentric (Schmid et al. 1979), and as such would be expected to show similar patterns of recombination. To provide useful context, we attempt to align the *L. vulgaris* linkage map with the *P. waltl* genome assembly (Brown et al. 2025). Surprisingly, given the challenges of aligning short, non‐coding sequences with a highly paralogous genome that diverged over 60 million years ago (Stewart and Wiens 2025), a useful comparison is possible and shows that genome‐level synteny between the two genera is highly conserved.

However, we are unable to confidently identify a sex‐determining region of this linkage map without using additional information. We do observe considerable variation in intra‐group marker density between the paternal and maternal maps, but this is not restricted to any one linkage group. In general, we show that *L. vulgaris* conforms to an extremely widespread pattern where male and female meiosis differ significantly, with males experiencing relatively more recombination near the telomeres and less recombination closer to the centromere of chromosomes (Sardell and Kirkpatrick 2020). Linkage group 5 varies slightly, as the telomeric region in which the Y‐linked markers cluster shows reduced paternal recombination. This could be interpreted as restricted recombination between the X and Y chromosomes; however, a similar phenomenon can also be observed in the autosomal linkage group 4.

If information about offspring sex is available and incorporated into the linkage map, we show that the sex‐linked region can readily be identified by the placement of sex‐linked markers. If such markers were not already known but offspring sex is known based on morphology, then they can be identified in the *ParentCall2* module of Lep‐MAP 3 (Rastas 2017). Alternatively, the knowledge of offspring sex could be used to conduct a sex‐association‐based analysis using a pipeline such as the one described above, or a tool such as RADsex (Feron et al. 2021). The latter approach is probably preferable as a dedicated tool allows for more flexibility in adjusting parameters and filtering candidate markers.

Brelsford et al. (2016) reported a significant excess of paternal‐specific SNPs on the linkage group corresponding to the Y‐chromosome of the European tree frog, *Hyla arborea* . In our study, however, we do not observe RAD markers bearing paternal‐specific SNPs to be more prevalent on a particular linkage group or form an obvious cluster within any linkage group. The likely explanation is that the sex‐determining region of *L. vulgaris* is simply too small to be observable with this methodology – especially given the enormous size of the overall genome and the consequently low per base pair recombination rate. In fact, linkage group 5 has slightly fewer than expected paternal RAD markers due to the random variation between groups obscuring the excess of paternal markers from within the tiny sex‐linked region.

As recombination frequency and the density of RAD markers will vary over the length of each chromosome, it is not possible to estimate the size of the Y‐linked region in *L. vulgaris* with any accuracy. However, we can identify orthologous loci for two Y‐linked presence/absence sequences within *P. waltl* chromosome 5, which corresponds to our linkage group 5. The orthologs are separated by just 27.3 Mbp, approximately 1.4% of the overall chromosome length. If this is reflective of the situation in the *L. vulgaris* Y‐linked region, it would explain the absence of an obvious cluster of paternal SNPs. A region this small would contain too few RAD markers to stand out against the genetic background, which will contain many other clusters of paternal (and maternal) specific SNPs.

It is somewhat unexpected that the non‐recombing region is not more prominent. A cytological study by Schmid et al. (1979) reported that, in male *L. vulgaris* , no chiasmata were observed in any region of the long arm of chromosome 5, which was hence identified as the Y‐chromosome, despite being largely homomorphic. While we do observe reduced paternal recombination at the end of linkage group 5 where the Y‐linked markers are placed, there is still a significant degree of recombination occurring within this region; the markers have not been collapsed to a single point on the paternal map. In addition, if the non‐recombing Y‐linked region covered the entirety of the long arm, we would expect a far greater density of paternal‐specific SNPs and markers than we observe. It is possible that *L. vulgaris* shows regional diversity in the structure and recombination frequency of the Y‐chromosome, as is noted in other amphibians, including *Hyla arborea* (Dufresnes et al. 2014) and the alpine newt *Ichthyosaura alpestris* (Herrero and López‐Fernández 1986). Such diversity may explain the discrepancy between the karyology performed on a population gathered near Ulm, Germany, and our linkage map generated from a Polish population collected in the vicinity of Kraków.

We conclude that, while linkage maps are of great benefit for locating previously discovered sex‐linked markers within a genome, their utility for identifying sex‐linked regions without a priori knowledge is strongly dependent on the size of the region of suppressed recombination. In species with very large genomes and small sex‐linked regions, the technique is unlikely to be successful. However, we show that an associative RADseq approach can still be highly effective even in these situations, especially when measures are taken to suppress the selection of markers with autosomal paralogs.

## Conflicts of Interest

The authors declare no conflicts of interest.

## Supporting information


Appendix S1.

Data S1.

## Data Availability

The full sex‐averaged, paternal and maternal linkage maps, and sequences of all markers placed on them are available as part of the accompanying Data S1. All scripts used in this study, as well as a pipeline describing their use, can be found in the GitHub repository https://github.com/Wielstra‐Lab/Lissotriton_RADseq_Y. This repository is archived in the associated Zenodo repository (10.5281/zenodo.13870461). The raw, demultiplexed RADseq reads from all samples used for generating sex‐associated candidate markers and constructing the linkage map are available as part of our NCBI submission (associated with Bioproject: PRJNA1118769)– see France et al. (2024).

## References

[men70019-bib-0001] An, J. , M. Lee , M.‐S. Min , M.‐H. Lee , and H. Lee . 2007. “A Molecular Genetic Approach for Species Identification of Mammals and Sex Determination of Birds in a Forensic Case of Poaching From South Korea.” Forensic Science International 167, no. 1: 59–61. 10.1016/j.forsciint.2005.12.031. 16460896

[men70019-bib-0002] Apuli, R.‐P. , C. Bernhardsson , B. Schiffthaler , et al. 2020. “Inferring the Genomic Landscape of Recombination Rate Variation in European Aspen ( *Populus tremula* ).” G3: Genes, Genomes, Genetics 10, no. 1: 299–309. 10.1534/g3.119.400504. 31744900 PMC6945010

[men70019-bib-0003] Babik, W. , W. Branicki , J. Crnobrnja‐Isailović , et al. 2005. “Phylogeography of Two European Newt Species—Discordance Between mtDNA and Morphology.” Molecular Ecology 14, no. 8: 2475–2491. 10.1111/j.1365-294X.2005.02605.x. 15969729

[men70019-bib-0004] Babik, W. , M. Marszałek , K. Dudek , et al. 2024. “Limited Evidence for Genetic Differentiation or Adaptation in Two Amphibian Species Across Replicated Rural–Urban Gradients.” Evolutionary Applications 17, no. 6: e13700. 10.1111/eva.13700. 38832082 PMC11146147

[men70019-bib-0005] Bachtrog, D. , J. E. Mank , C. L. Peichel , et al. 2014. “Sex Determination: Why So Many Ways of Doing It?” PLoS Biology 12, no. 7: e1001899. 10.1371/journal.pbio.1001899. 24983465 PMC4077654

[men70019-bib-0006] Bayona‐Vásquez, N. J. , T. C. Glenn , T. J. Kieran , et al. 2019. “Adapterama III: Quadruple‐Indexed, Double/Triple‐Enzyme RADseq Libraries (2RAD/3RAD).” PeerJ 7: e7724. 10.7717/peerj.7724. 31616583 PMC6791345

[men70019-bib-0007] Brelsford, A. , C. Dufresnes , and N. Perrin . 2016. “High‐Density Sex‐Specific Linkage Maps of a European Tree Frog ( *Hyla arborea* ) Identify the Sex Chromosome Without Information on Offspring Sex.” Heredity 116, no. 2: 177–181. 10.1038/hdy.2015.83. 26374238 PMC4806884

[men70019-bib-0008] Brelsford, A. , G. Lavanchy , R. Sermier , A. Rausch , and N. Perrin . 2017. “Identifying Homomorphic Sex Chromosomes From Wild‐Caught Adults With Limited Genomic Resources.” Molecular Ecology Resources 17, no. 4: 752–759. 10.1111/1755-0998.12624. 27790846

[men70019-bib-0009] Brown, T. , K. Mishra , A. Elewa , et al. 2025. “Chromosome‐Scale Genome Assembly Reveals How Repeat Elements Shape Non‐Coding RNA Landscapes Active During Newt Limb Regeneration.” Cell Genomics 5: 100761. 10.1016/j.xgen.2025.100761. 39874962 PMC11872487

[men70019-bib-0010] Camacho, C. , G. Coulouris , V. Avagyan , et al. 2009. “BLAST+: Architecture and Applications.” BMC Bioinformatics 10, no. 1: 421. 10.1186/1471-2105-10-421. 20003500 PMC2803857

[men70019-bib-0011] Catchen, J. , P. A. Hohenlohe , S. Bassham , A. Amores , and W. A. Cresko . 2013. “Stacks: An Analysis Tool Set for Population Genomics.” Molecular Ecology 22, no. 11: 3124–3140. 10.1111/mec.12354. 23701397 PMC3936987

[men70019-bib-0012] Charlesworth, D. , B. Charlesworth , and G. Marais . 2005. “Steps in the Evolution of Heteromorphic Sex Chromosomes.” Heredity 95, no. 2: 118–128. 10.1038/sj.hdy.6800697. 15931241

[men70019-bib-0013] Cortez, D. , R. Marin , D. Toledo‐Flores , et al. 2014. “Origins and Functional Evolution of Y Chromosomes Across Mammals.” Nature 508, no. 7497: 488–493. 10.1038/nature13151. 24759410

[men70019-bib-0014] Curzon, A. Y. , A. Shirak , T. Zak , et al. 2021. “All‐Male Production by Marker‐Assisted Selection for Sex Determining Loci of Admixed *Oreochromis niloticus* and *Oreochromis aureus* Stocks.” Animal Genetics 52, no. 3: 361–364. 10.1111/age.13057. 33740255

[men70019-bib-0015] Danecek, P. , A. Auton , G. Abecasis , et al. 2011. “The Variant Call Format and VCFtools.” Bioinformatics 27, no. 15: 2156–2158. 10.1093/bioinformatics/btr330. 21653522 PMC3137218

[men70019-bib-0016] Danecek, P. , J. K. Bonfield , J. Liddle , et al. 2021. “Twelve Years of SAMtools and BCFtools.” GigaScience 10, no. 2: giab008. 10.1093/gigascience/giab008. 33590861 PMC7931819

[men70019-bib-0017] Darolti, I. , A. E. Wright , B. A. Sandkam , et al. 2019. “Extreme Heterogeneity in Sex Chromosome Differentiation and Dosage Compensation in Livebearers.” Proceedings of the National Academy of Sciences 116, no. 38: 19031–19036. 10.1073/pnas.1905298116. PMC6754558 31484763

[men70019-bib-0018] De, R. , B. D. Joshi , M. Shukla , P. Pandey , R. Singh , and S. P. Goyal . 2019. “Understanding Predation Behaviour of the Tiger ( *Panthera tigris tigris* ) in Ranthambore Tiger Reserve, Rajasthan, India: Use of Low‐Cost Gel Based Molecular Sexing of Prey Hairs From Scats.” Conservation Genetics Resources 11, no. 1: 97–104. 10.1007/s12686-017-0963-2.

[men70019-bib-0019] Dufresnes, C. , A. Brelsford , and N. Perrin . 2014. “First‐Generation Linkage Map for The European Tree frog ( *Hyla arborea* ) With Utility in Congeneric Species.” BMC Research Notes 7, no. 1: 850. 10.1186/1756-0500-7-850. 25430653 PMC4258042

[men70019-bib-0020] Dufresnes, C. , and P.‐A. Crochet . 2022. “Sex Chromosomes as Supergenes of Speciation: Why Amphibians Defy the Rules?” Philosophical Transactions of the Royal Society, B: Biological Sciences 377, no. 1856: 20210202. 10.1098/rstb.2021.0202. PMC9189495 35694748

[men70019-bib-0021] Ellegren, H. 2010. “Evolutionary Stasis: The Stable Chromosomes of Birds.” Trends in Ecology & Evolution 25, no. 5: 283–291. 10.1016/j.tree.2009.12.004. 20363047

[men70019-bib-0022] Ezaz, T. , S. D. Sarre , D. O'Meally , J. A. Marshall Graves , and A. Georges . 2010. “Sex Chromosome Evolution in Lizards: Independent Origins and Rapid Transitions.” Cytogenetic and Genome Research 127, no. 2–4: 249–260. 10.1159/000300507. 20332599

[men70019-bib-0023] Feron, R. , Q. Pan , M. Wen , et al. 2021. “RADSex: A Computational Workflow to Study Sex Determination Using Restriction Site‐Associated DNA Sequencing Data.” Molecular Ecology Resources 21, no. 5: 1715–1731. 10.1111/1755-0998.13360. 33590960 PMC8589568

[men70019-bib-0024] France, J. , W. Babik , K. Dudek , M. Marszałek , and B. Wielstra . 2024. Lissotriton Vulgaris Linkage Mapping and Y‐Chromosome Identification (PRJNA1118769). NCBI.

[men70019-bib-0025] Fridolfsson, A.‐K. , and H. Ellegren . 1999. “A Simple and Universal Method for Molecular Sexing of Non‐Ratite Birds.” Journal of Avian Biology 30, no. 1: 116–121. 10.2307/3677252.

[men70019-bib-0026] Gamble, T. , J. Coryell , T. Ezaz , J. Lynch , D. P. Scantlebury , and D. Zarkower . 2015. “Restriction Site‐Associated DNA Sequencing (RAD‐Seq) Reveals an Extraordinary Number of Transitions Among Gecko Sex‐Determining Systems.” Molecular Biology and Evolution 32, no. 5: 1296–1309. 10.1093/molbev/msv023. 25657328

[men70019-bib-0027] Gamble, T. , and D. Zarkower . 2014. “Identification of Sex‐Specific Molecular Markers Using Restriction Site‐Associated DNA Sequencing.” Molecular Ecology Resources 14, no. 5: 902–913. 10.1111/1755-0998.12237. 24506574

[men70019-bib-0028] Gregory, R. T. 2024. “Animal Genome Size Database.” https://www.genomesize.com/.

[men70019-bib-0029] Herrero, P. , and C. López‐Fernández . 1986. “The Meiotic System of Iberian Species of the Genus *Triturus* (Amphibia: Caudata).” Caryologia 39, no. 3–4: 385–395. 10.1080/00087114.1986.10797801.

[men70019-bib-0030] Hime, P. M. , J. T. Briggler , J. S. Reece , and D. W. Weisrock . 2019. “Genomic Data Reveal Conserved Female Heterogamety in Giant Salamanders With Gigantic Nuclear Genomes.” G3: Genes, Genomes, Genetics 9, no. 10: 3467–3476. 10.1534/g3.119.400556. 31439718 PMC6778777

[men70019-bib-0031] Hrovatin, K. , and T. Kunej . 2018. “Genetic Sex Determination Assays in 53 Mammalian Species: Literature Analysis and Guidelines for Reporting Standardization.” Ecology and Evolution 8, no. 2: 1009–1018. 10.1002/ece3.3707. 29375774 PMC5773321

[men70019-bib-0032] Hu, Q. , C. Chang , Q. Wang , et al. 2019. “Genome‐Wide RAD Sequencing to Identify a Sex‐Specific Marker in Chinese Giant Salamander *Andrias davidianus* .” BMC Genomics 20, no. 1: 415. 10.1186/s12864-019-5771-5. 31122206 PMC6533744

[men70019-bib-0033] Hu, Q. , Y. Liu , X. Liao , et al. 2021. “A High‐Density Genetic Map Construction and Sex‐Related Loci Identification in Chinese Giant Salamander.” BMC Genomics 22, no. 1: 230. 10.1186/s12864-021-07550-0. 33794798 PMC8017863

[men70019-bib-0034] Intha, N. , and P. Chaiprasart . 2020. “Development of Molecular Markers for Sex Determination in *Phoenix dactylifera L* .” Acta Horticulturae 1299: 425–432. 10.17660/ActaHortic.2020.1299.64.

[men70019-bib-0035] Johnson, N. A. , and J. Lachance . 2012. “The Genetics of Sex Chromosomes: Evolution and Implications for Hybrid Incompatibility.” Annals of the New York Academy of Sciences 1256, no. 1: E1–E22. 10.1111/j.1749-6632.2012.06748.x. 23025408 PMC3509754

[men70019-bib-0036] Keinath, M. C. , N. Timoshevskaya , V. A. Timoshevskiy , S. R. Voss , and J. J. Smith . 2018. “Miniscule Differences Between Sex Chromosomes in the Giant Genome of A Salamander.” Scientific Reports 8, no. 1: 17882. 10.1038/s41598-018-36209-2. 30552368 PMC6294749

[men70019-bib-0037] Kitano, J. , S. Ansai , Y. Takehana , and Y. Yamamoto . 2024. “Diversity and Convergence of Sex‐Determination Mechanisms in Teleost Fish.” Annual Review of Animal Biosciences 12: 233–259. 10.1146/annurev-animal-021122-113935. 37863090

[men70019-bib-0038] Lee, B.‐Y. , M.‐S. Kim , B.‐S. Choi , et al. 2019. “Construction of High‐Resolution RAD‐Seq Based Linkage Map, Anchoring Reference Genome, and QTL Mapping of the Sex Chromosome in the Marine Medaka *Oryzias melastigma* .” G3: Genes, Genomes, Genetics 9, no. 11: 3537–3545. 10.1534/g3.119.400708. 31530635 PMC6829124

[men70019-bib-0039] Lee, S.‐I. , and N.‐S. Kim . 2014. “Transposable Elements and Genome Size Variations in Plants.” Genomics & Informatics 12, no. 3: 87–97. 10.5808/GI.2014.12.3.87. 25317107 PMC4196380

[men70019-bib-0040] Litvinchuk, S. N. , J. M. Rosanov , and L. J. Borkin . 2007. “Correlations of Geographic Distribution and Temperature of Embryonic Development With the Nuclear DNA Content in the Salamandridae (Urodela, Amphibia).” Genome 50, no. 4: 333–342. 10.1139/G07-010. 17546091

[men70019-bib-0041] Ma, W.‐J. , and M. Rovatsos . 2022. “Sex Chromosome Evolution: The Remarkable Diversity in the Evolutionary Rates and Mechanisms.” Journal of Evolutionary Biology 35, no. 12: 1581–1588. 10.1111/jeb.14119. 36455930

[men70019-bib-0042] Mars, J. , S. Koster , W. Babik , et al. 2025. “Phylogenomics Yields New Systematic and Taxonomical Insights for *Lissotriton* Newts, a Genus With A Strong Legacy of Introgressive Hybridization.” Molecular Phylogenetics and Evolution 204: 108282. 10.1016/j.ympev.2024.108282. 39746557

[men70019-bib-0043] Meilink, W. R. M. , M. C. de Visser , A. Theodoropoulos , M. Fahrbach , and B. Wielstra . 2025. “Determining Zygosity With Multiplex Kompetitive Allele‐Specific PCR (mxKASP) Genotyping.” Ecology and Evolution 15, no. 6: e71642. 10.1002/ece3.71642. 40546918 PMC12181748

[men70019-bib-0044] Miller, M. R. , J. P. Dunham , A. Amores , W. A. Cresko , and E. A. Johnson . 2007. “Rapid and Cost‐Effective Polymorphism Identification and Genotyping Using Restriction Site Associated DNA (RAD) Markers.” Genome Research 17, no. 2: 240–248. 10.1101/gr.5681207. 17189378 PMC1781356

[men70019-bib-0045] Miralles, A. , J. Secondi , M. Pabijan , W. Babik , C. Lemaire , and P.‐A. Crochet . 2024. “Inconsistent Estimates of Hybridization Frequency in Newts Revealed by SNPs and Microsatellites.” Conservation Genetics 25, no. 1: 215–225. 10.1007/s10592-023-01556-9.

[men70019-bib-0046] Miura, I. 2017. “Sex Determination and Sex Chromosomes in Amphibia.” Sexual Development 11, no. 5–6: 298–306. 10.1159/000485270. 29241181

[men70019-bib-0047] Niedzicka, M. , K. Dudek , A. Fijarczyk , P. Zieliński , and W. Babik . 2017. “Linkage Map of *Lissotriton* Newts Provides Insight Into the Genetic Basis of Reproductive Isolation.” G3: Genes, Genomes, Genetics 7, no. 7: 2115–2124. 10.1534/g3.117.041178. 28500054 PMC5499121

[men70019-bib-0048] Pabijan, M. , P. Zieliński , K. Dudek , M. Stuglik , and W. Babik . 2017. “Isolation and Gene Flow in a Speciation Continuum in Newts.” Molecular Phylogenetics and Evolution 116: 1–12. 10.1016/j.ympev.2017.08.003. 28797693

[men70019-bib-0049] Palmer, D. H. , T. F. Rogers , R. Dean , and A. E. Wright . 2019. “How to Identify Sex Chromosomes and Their Turnover.” Molecular Ecology 28, no. 21: 4709–4724. 10.1111/mec.15245. 31538682 PMC6900093

[men70019-bib-0050] Paris, J. R. , J. R. Stevens , and J. M. Catchen . 2017. “Lost in Parameter Space: A Road Map for Stacks.” Methods in Ecology and Evolution 8, no. 10: 1360–1373. 10.1111/2041-210X.12775.

[men70019-bib-0051] Payseur, B. A. , D. C. Presgraves , and D. A. Filatov . 2018. “Introduction: Sex Chromosomes and Speciation.” Molecular Ecology 27, no. 19: 3745–3748. 10.1111/mec.14828. 30086196 PMC6179907

[men70019-bib-0052] Pellicer, J. , and I. J. Leitch . 2020. “The Plant DNA C‐Values Database (release 7.1): An Updated Online Repository of Plant Genome Size Data for Comparative Studies.” New Phytologist 226, no. 2: 301–305. 10.1111/nph.16261. 31608445

[men70019-bib-0053] Peng, W. , J. Xu , Y. Zhang , et al. 2016. “An Ultra‐High Density Linkage Map and QTL Mapping for Sex and Growth‐Related Traits of Common Carp (*Cyprinus carpio*).” Scientific Reports 6, no. 1: 26693. 10.1038/srep26693. 27225429 PMC4880943

[men70019-bib-0054] Purcell, J. , A. Brelsford , Y. Wurm , N. Perrin , and M. Chapuisat . 2014. “Convergent Genetic Architecture Underlies Social Organization in Ants.” Current Biology 24, no. 22: 2728–2732. 10.1016/j.cub.2014.09.071. 25455032

[men70019-bib-0055] Rafati, N. , J. Chen , A. Herpin , et al. 2020. “Reconstruction of the Birth of a Male Sex Chromosome Present in Atlantic Herring.” Proceedings of the National Academy of Sciences 117, no. 39: 24359–24368. 10.1073/pnas.2009925117. PMC7533707 32938798

[men70019-bib-0056] Rastas, P. 2017. “Lep‐MAP3: Robust Linkage Mapping Even for Low‐Coverage Whole Genome Sequencing Data.” Bioinformatics 33, no. 23: 3726–3732. 10.1093/bioinformatics/btx494. 29036272

[men70019-bib-0057] Renner, S. S. 2014. “The Relative and Absolute Frequencies of Angiosperm Sexual Systems: Dioecy, Monoecy, Gynodioecy, and an Updated Online Database.” American Journal of Botany 101, no. 10: 1588–1596. 10.3732/ajb.1400196. 25326608

[men70019-bib-0058] Rochette, N. C. , A. G. Rivera‐Colón , and J. M. Catchen . 2019. “Stacks 2: Analytical Methods for Paired‐End Sequencing Improve RADseq‐Based Population Genomics.” Molecular Ecology 28, no. 21: 4737–4754. 10.1111/mec.15253. 31550391

[men70019-bib-0059] Sambrook, J. , and D. W. Russell . 2001. Molecular Cloning: A Laboratory Manual. CSHL Press.

[men70019-bib-0060] Sardell, J. M. , and M. Kirkpatrick . 2020. “Sex Differences in the Recombination Landscape.” American Naturalist 195, no. 2: 361–379. 10.1086/704943. PMC7537610 32017625

[men70019-bib-0061] Schmid, M. , J. Olert , and C. Klett . 1979. “Chromosome Banding in Amphibia.” Chromosoma 71, no. 1: 29–55. 10.1007/BF00426365.

[men70019-bib-0062] Sparreboom, M. 2014. Salamanders of the Old World: The Salamanders of Europe, Asia and Northern Africa. KNNV Publishing. 10.1163/9789004285620.

[men70019-bib-0063] Stewart, A. A. , and J. J. Wiens . 2025. “A Time‐Calibrated Salamander Phylogeny Including 765 Species and 503 Genes.” Molecular Phylogenetics and Evolution 204: 108272. 10.1016/j.ympev.2024.108272. 39681150

[men70019-bib-0064] Sun, C. , D. B. Shepard , R. A. Chong , et al. 2012. “LTR Retrotransposons Contribute to Genomic Gigantism in Plethodontid Salamanders.” Genome Biology and Evolution 4, no. 2: 168–183. 10.1093/gbe/evr139. 22200636 PMC3318908

[men70019-bib-0065] Trenkel, V. M. , P. Boudry , V. Verrez‐Bagnis , and P. Lorance . 2020. “Methods for Identifying and Interpreting Sex‐Linked SNP Markers and Carrying Out Sex Assignment: Application to Thornback Ray ( *Raja clavata* ).” Molecular Ecology Resources 20, no. 6: 1610–1619. 10.1111/1755-0998.13225. 32657500

[men70019-bib-0066] Untergasser, A. , I. Cutcutache , T. Koressaar , et al. 2012. “Primer3—New Capabilities and Interfaces.” Nucleic Acids Research 40, no. 15: e115. 10.1093/nar/gks596. 22730293 PMC3424584

[men70019-bib-0067] van Doorn, G. S. , and M. Kirkpatrick . 2010. “Transitions Between Male and Female Heterogamety Caused by Sex‐Antagonistic Selection.” Genetics 186, no. 2: 629–645. 10.1534/genetics.110.118596. 20628036 PMC2954476

[men70019-bib-0068] Vidya, T. N. C. , V. R. Kumar , C. Arivazhagan , and R. Sukumar . 2003. “Application of Molecular Sexing To Free‐Ranging Asian Elephant (*Elephas maximus*) Populations in Southern India.” Current Science 85, no. 7: 1074–1077.

[men70019-bib-0069] Wickbom, T. 1945. “Cytological Studies on Dipnoi, Urodela, Anura, and Emys.” Hereditas 31, no. 3–4: 241–346. 10.1111/j.1601-5223.1945.tb02756.x. 21021072

[men70019-bib-0070] Wielstra, B. , D. Canestrelli , M. Cvijanović , et al. 2018. “The Distributions of the Six Species Constituting the Smooth Newt Species Complex (*Lissotriton vulgaris sensu lato* and *L. montandoni*) – An Addition to the New Atlas of Amphibians and Reptiles of Europe.” Amphibia‐Reptilia 39, no. 2: 252–259. 10.1163/15685381-17000128.

[men70019-bib-0071] Wright, A. E. , R. Dean , F. Zimmer , and J. E. Mank . 2016. “How to Make a Sex Chromosome.” Nature Communications 7, no. 1: 12087. 10.1038/ncomms12087. PMC4932193 27373494

[men70019-bib-0072] Yi, X. , P. Kemppainen , and J. Merilä . 2024. “SLRfinder: A Method to Detect Candidate Sex‐Linked Regions With Linkage Disequilibrium Clustering.” Molecular Ecology Resources 24, no. 6: e13985. 10.1111/1755-0998.13985. 38850116

[men70019-bib-0073] Yoshida, K. , T. Makino , K. Yamaguchi , et al. 2014. “Sex Chromosome Turnover Contributes to Genomic Divergence Between Incipient Stickleback Species.” PLoS Genetics 10, no. 3: e1004223. 10.1371/journal.pgen.1004223. 24625862 PMC3953013

[men70019-bib-0074] Zboźeń, J. , and J. Rafiński . 1993. “A Comparative Study of Mitotic and Meiotic Chromosomes of the Montandon's Newt, *Triturus montandoni* (Urodela: Salamandridae) From Poland and Rumania.” Genetica 88, no. 1: 69. 10.1007/BF02424453.

