# Supplementary material for: Linkage Mapping vs. Association: A Comparison of Two RADseq‐Based Approaches to Identify Markers for Homomorphic Sex Chromosomes in Large Genomes
Source: Mol Ecol Resour. 2025 Jul 24;25(7):e70019. doi: 10.1111/1755-0998.70019 (PMC12415805; doi:10.1111/1755-0998.70019)
Supplement: Supplementary file 1 — Appendix S1. [file MEN-25-e70019-s001.pdf]

MOLECULAR ECOLOGY RESOURCES

Supplemental Information for:

Linkage mapping vs Association: A comparison of two RADseq-based approaches to identify markers for homomorphic sex chromosomes in large genomes.

James France, Wiesław Babik, Katarzyna Dudek, Marzena Marszałek, Ben Wielstra

Table of Contents:

**Figure S1: Screening in *L. vulgaris***	Page 2
**Figure S2: Screening in *L. montandoni* and *L. helveticus***	Page 3
**Figure S3: Screening in other *Lissotriton* species**	Page 4
**Figure S4: Validation in *L. vulgaris***	Page 5
**Figure S5: Multiplex PCR validation in RADseq adults**	Page 6
**Figure S6: Multiplex PCR in *L. vulgaris* and *L. montandoni***	Page 7
**Figure S7: Paternal Linkage map**	Page 8
**Figure S8: Maternal Linkage map**	Page 9
**Figure S9: Linkage map incorporating RADsex markers**	Page 10
**Figure S10: Linkage map incorporating ParentCall markers**	Page 11
**Table S1: Linkage map statistics**	Page 12
**Table S2: Distribution of *L. vulgaris* markers in *P. waltl***	Page 12
**Table S3: Primer sequences**	Page 13
**Table S4: Sample information**	Page 14

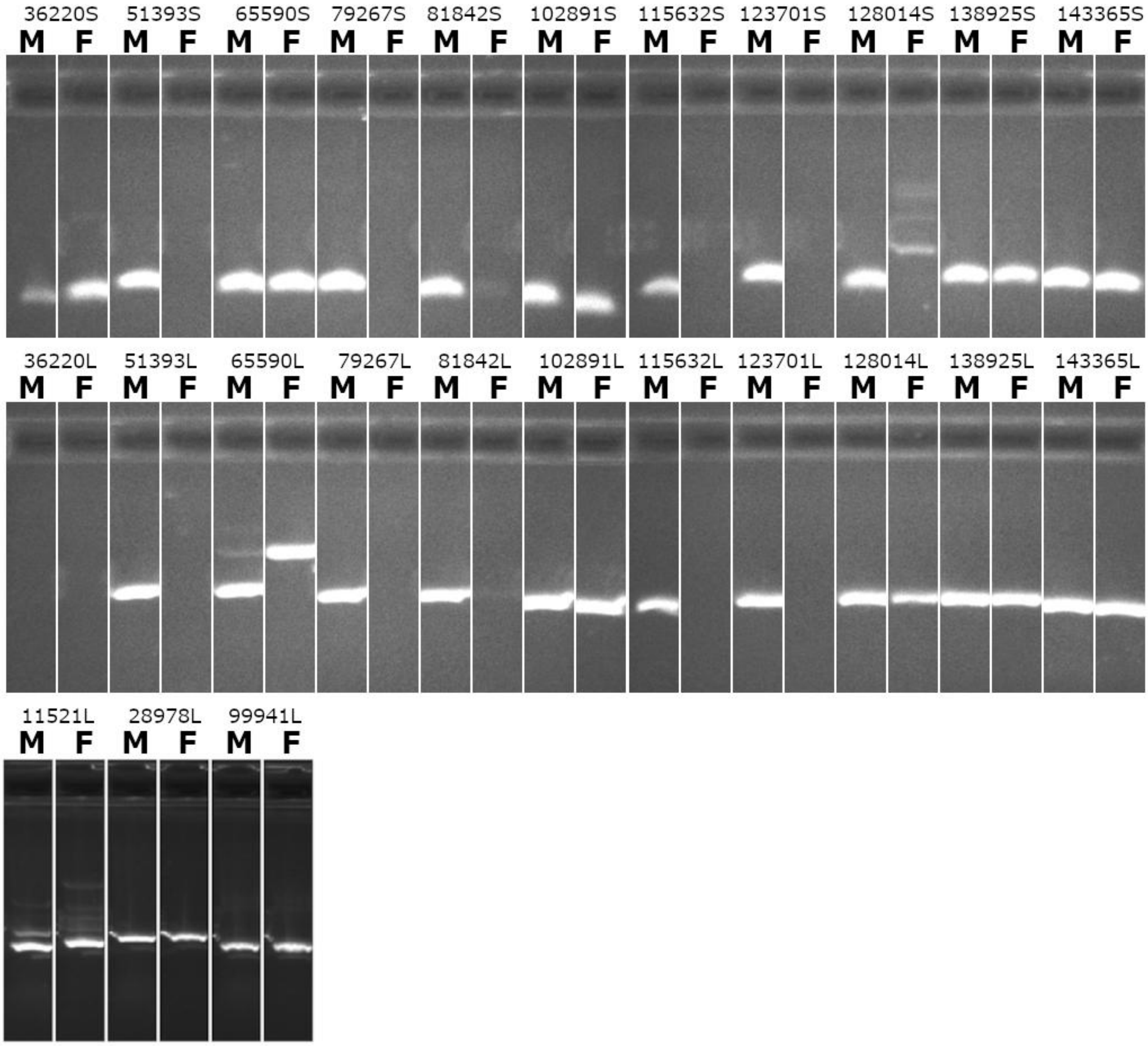

Figure S1: PCR screening of 25 primer pairs designed for candidate Y-linked markers for male specific amplification in *L. vulgaris*. Label M indicates the male sample and label F indicates female. Markers are indicated by number followed by either S (for primer pairs designed for the short product – c.a. 100 bp) or L (for primer pairs designed for the long product – c.a. 200 bp). 10 primer pairs, and 5 markers showed amplification only in the male sample. One further pair, LvY-128014-short showed strong amplification in the male and only weak amplification in the female. A single primer pair, LvY-36220-long, failed to amplify in either sample. The other 25 primer pairs amplified in both the male and female samples.

MOLECULAR ECOLOGY RESOURCES

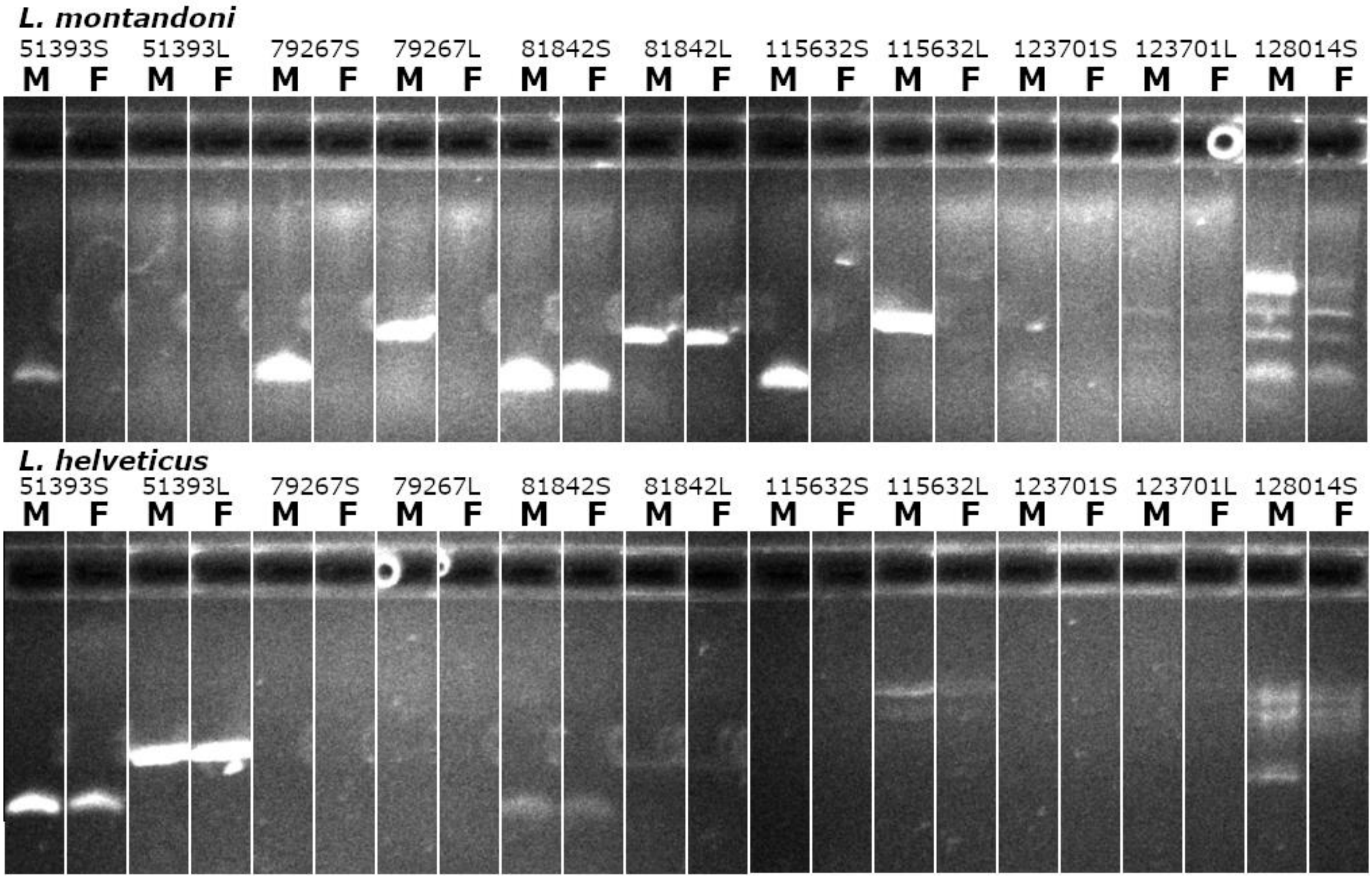

Figure S2: PCR screening of the 11 primer pairs showing successful male-specific amplification in *L. vulgaris*, *in L. montandoni* and *L. helveticus*. Label M indicates the male sample and label F indicates female. Markers are indicated by number followed by either S (for primer pairs designed for the short product – c.a. 100 bp) or L (for primer pairs designed for the long product – c.a. 200 bp). Five primer pairs, designed for three marker sequences, show male specific amplification in *L. montandoni*. Only two primer pairs show strong amplification in *L. helveticus*, and no male specificity is observed.

MOLECULAR ECOLOGY
RESOURCES

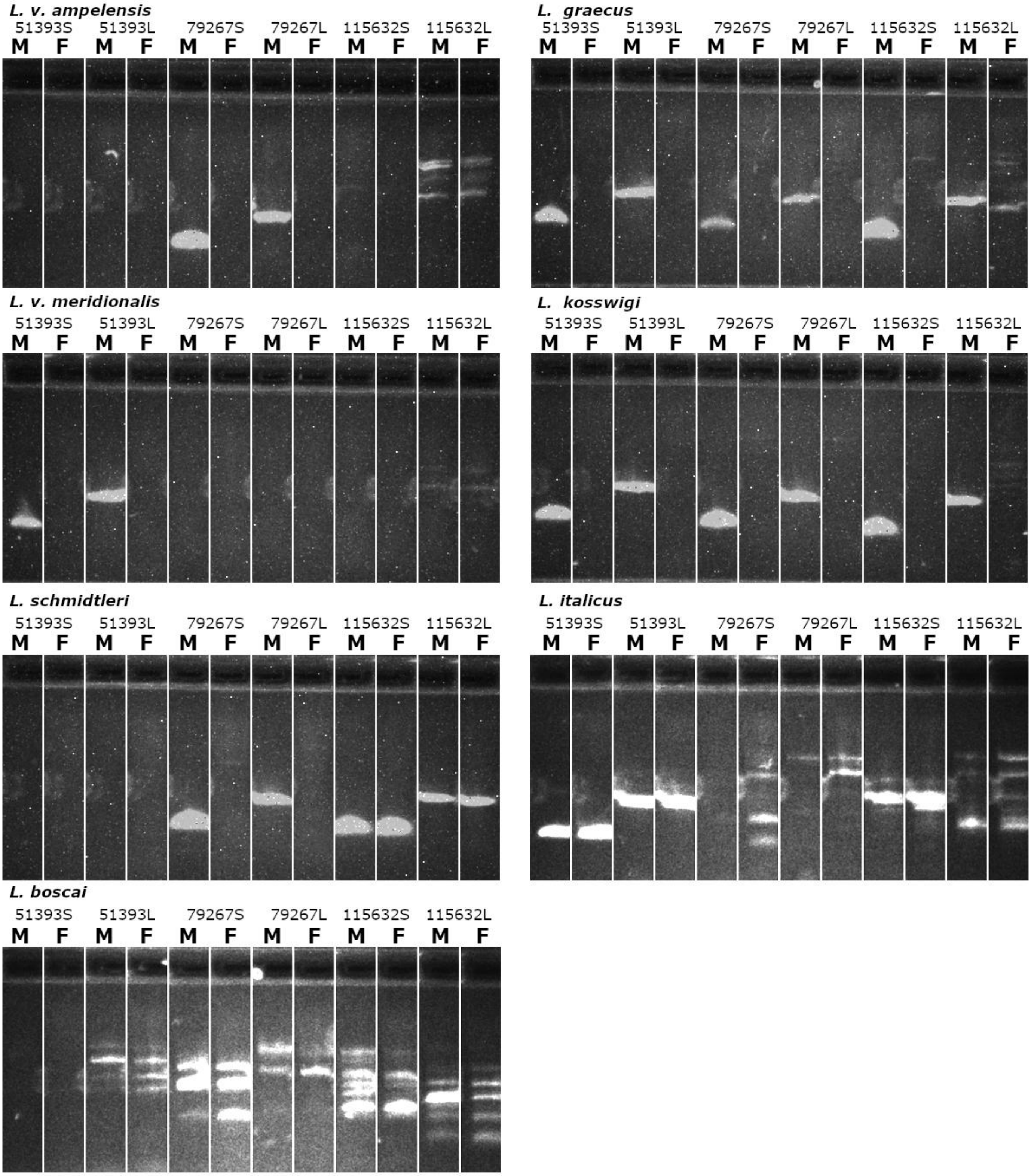

Figure S3: PCR screening in seven further *Lissotriton* taxa, using the primer pairs for the three markers that show male specificity in *L. montandoni*. For the five taxa in the L. vulgaris species complex, at least one marker shows male-specific amplification. In the two more distantly related species (*L. italicus* and *L. boscai*) no male-specific amplification is observed, and multiple non-target bands appear.

MOLECULAR ECOLOGY
RESOURCES

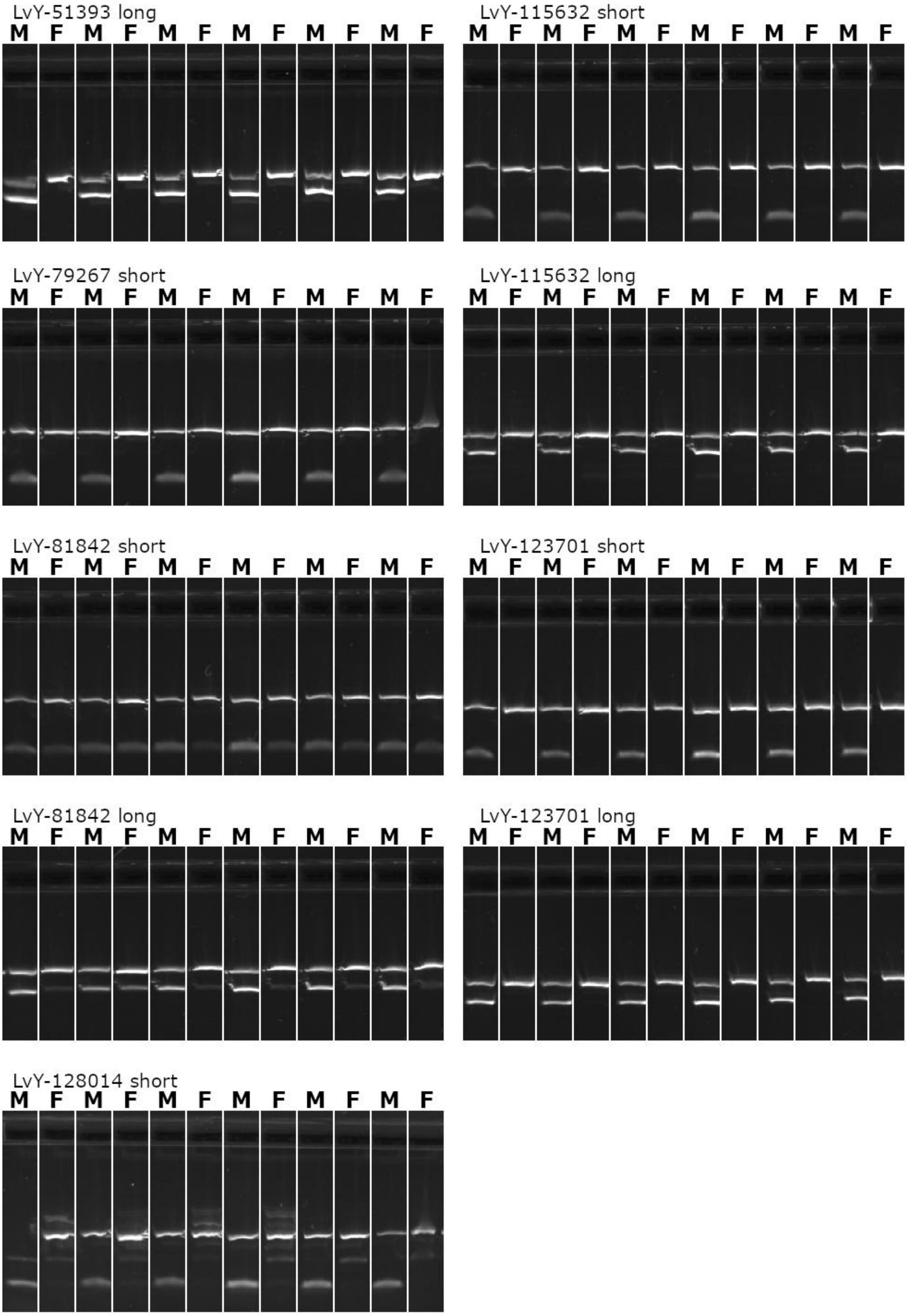

Figure S4: PCR validation of primer pairs showing male specificity in *L. vulgaris*, in a 12 individual panel (six male, six female, not including the two individuals previously used for screening, labelled as used for 'Validation' in table S4). Primers amplifying CDK-17 were included as a control, and the resultant product appears above the test bands in all cases due to greater length (517 bp compared to 100-250 bp). Both primer pairs designed for LvY-81842 appear to amplify in their product in females and thus fail validation. All other primer pairs are validated as male specific.

MOLECULAR ECOLOGY
RESOURCES

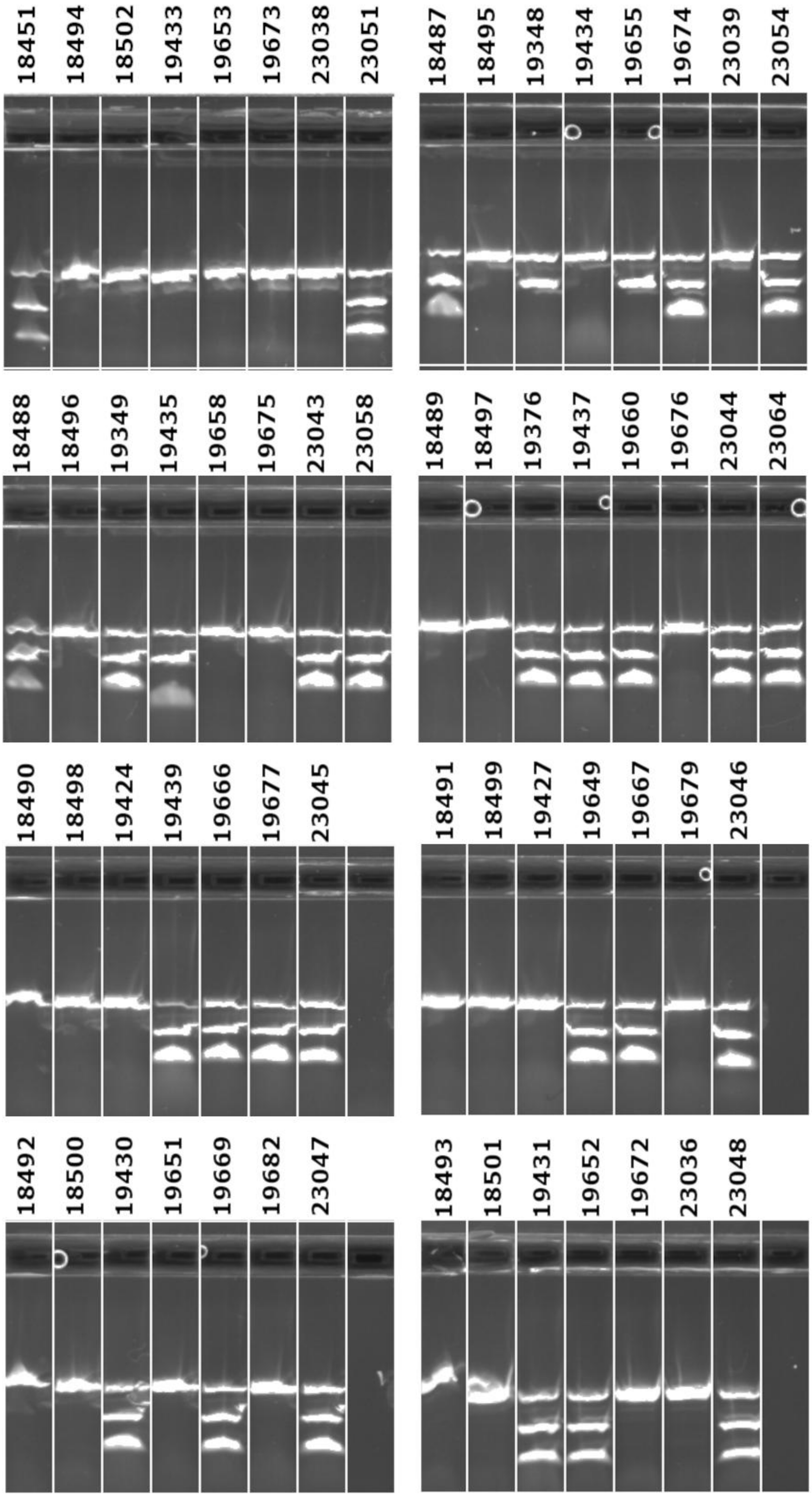

Figure S5: Validation of the multiplex PCR protocol (LvY-79267-Long and LvY-51393-Short with CDK-17 as a control) in the 30 known-sex adult *L. vulgaris* used for identification of sex-associated markers via RADseq. The PCR protocol results in accurate sex identification in all individuals.

MOLECULAR ECOLOGY
RESOURCES

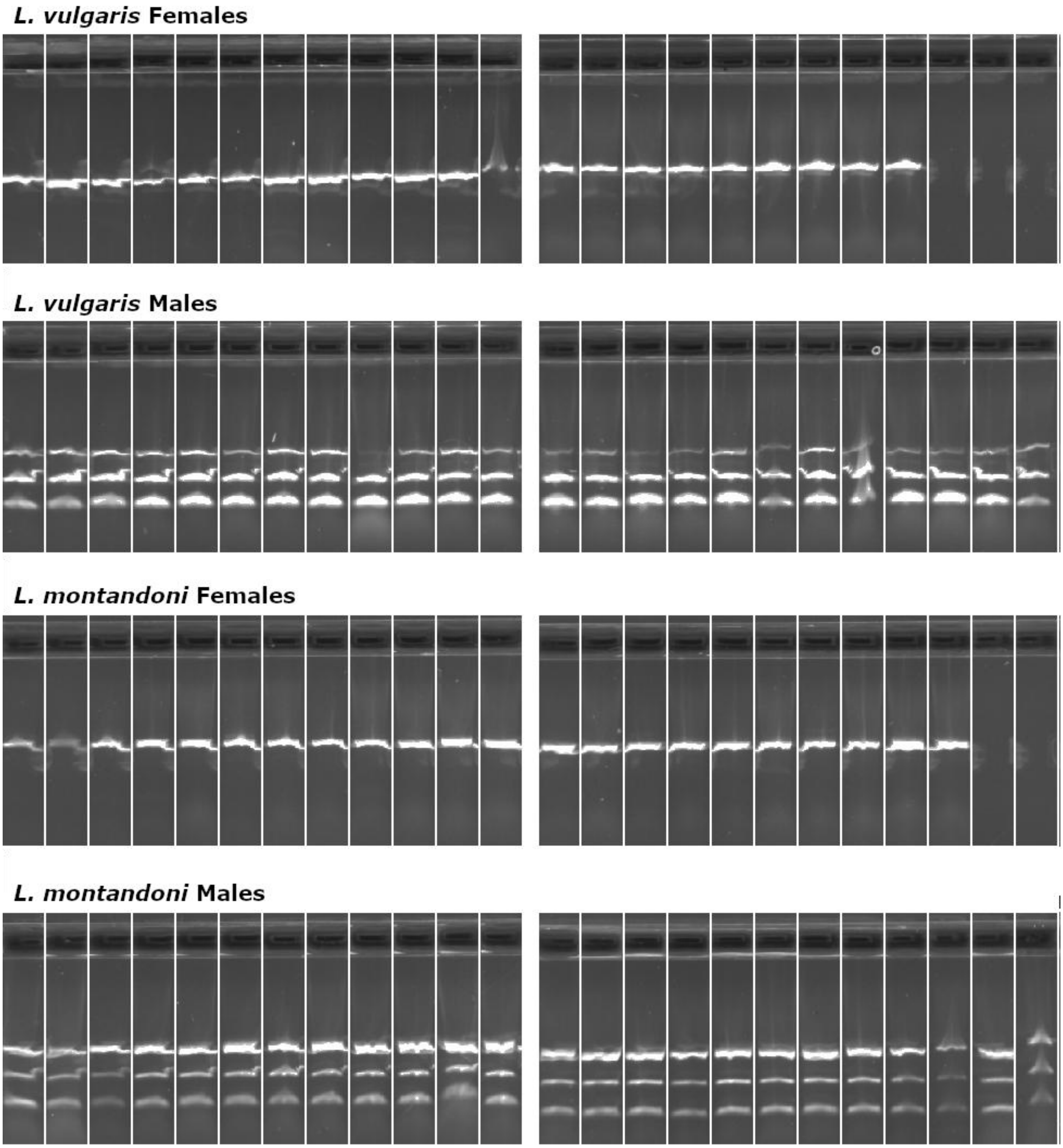

Figure S6: Validation of the multiplex PCR protocol (LvY-79267-Long and LvY-51393-Short with CDK-17 as a control) in larger sample sets of *L. vulgaris* (24 males and 21 females) and *L. montandoni* (24 males and 22 females) using samples denoted as used for 'PCR validation' in table S4, including previous validation and screening samples. The multiplex PCR protocol accurately identifies sex in all 91 individuals.

MOLECULAR ECOLOGY RESOURCES

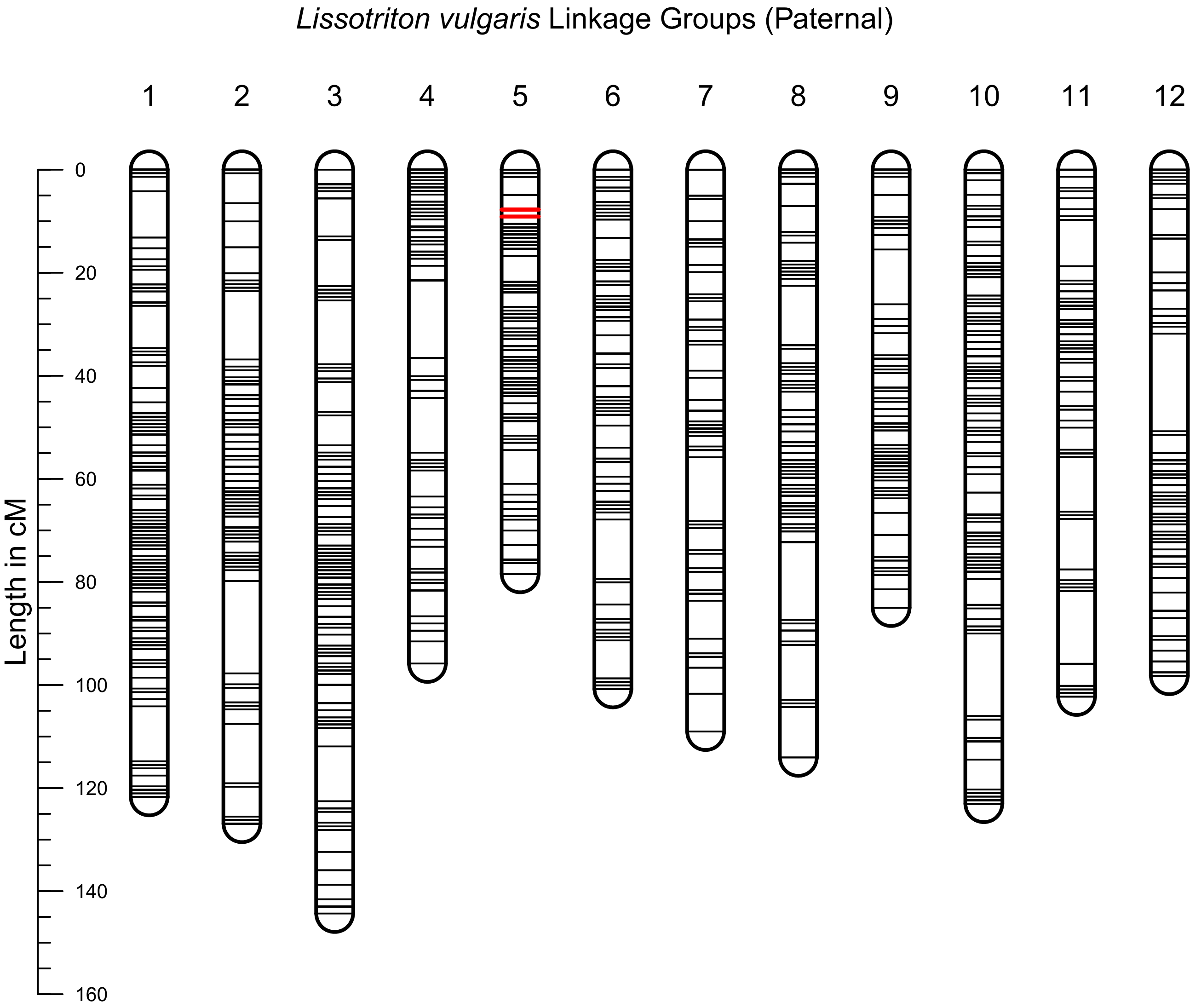

Figure S7: The paternal *L. vulgaris* linkage map, displaying 7,484 RAD markers across 12 linkage groups, including 32 Y-linked markers highlighted in red on linkage group 5. Groups are ordered according to the length of the corresponding group in the sex averaged map.

MOLECULAR ECOLOGY RESOURCES

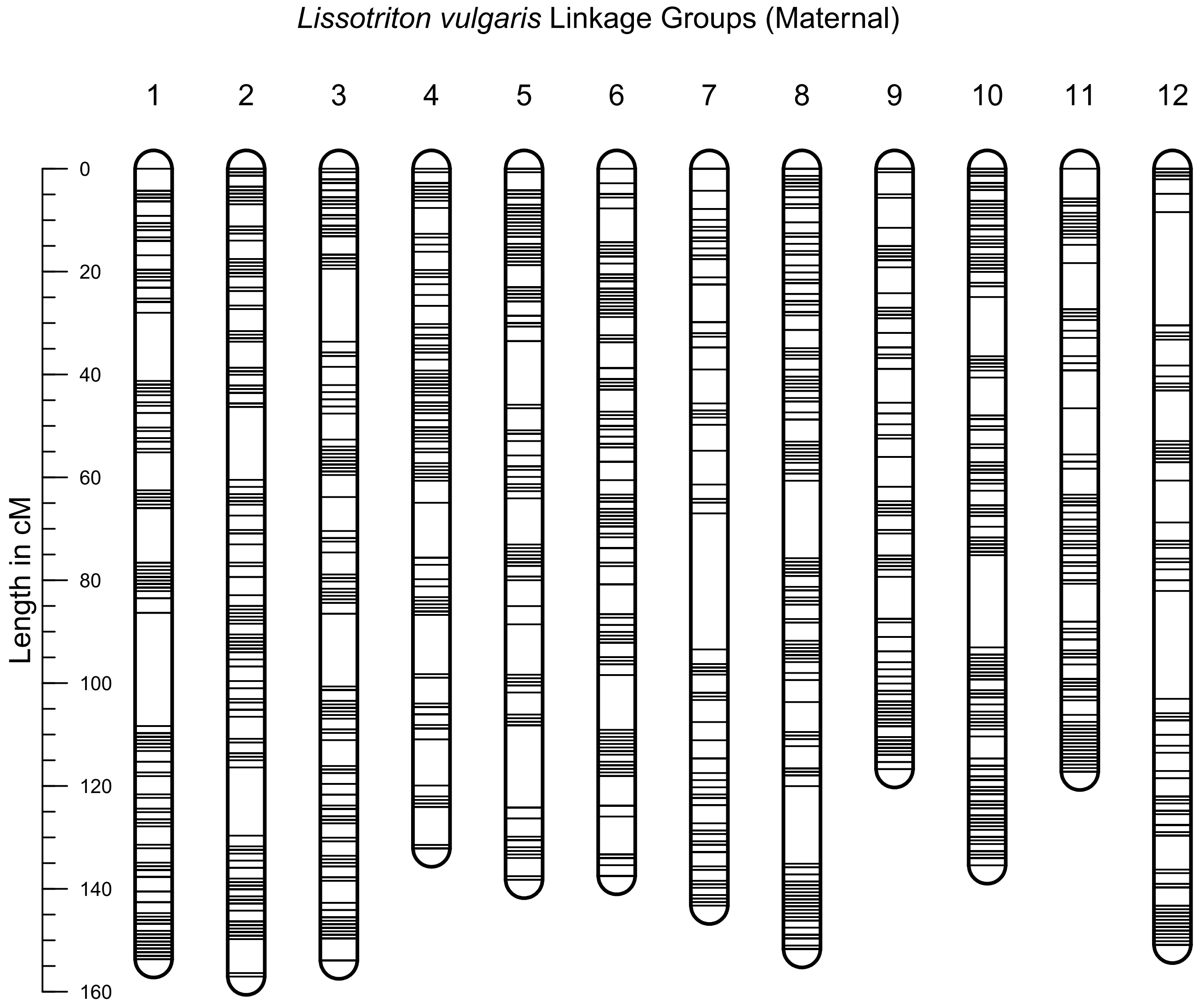

Figure S8: The maternal *L. vulgaris* linkage map, displaying 7,452 RAD markers across 12 linkage groups. Groups are ordered according to the length of the corresponding group in the sex averaged map.

MOLECULAR ECOLOGY
RESOURCES

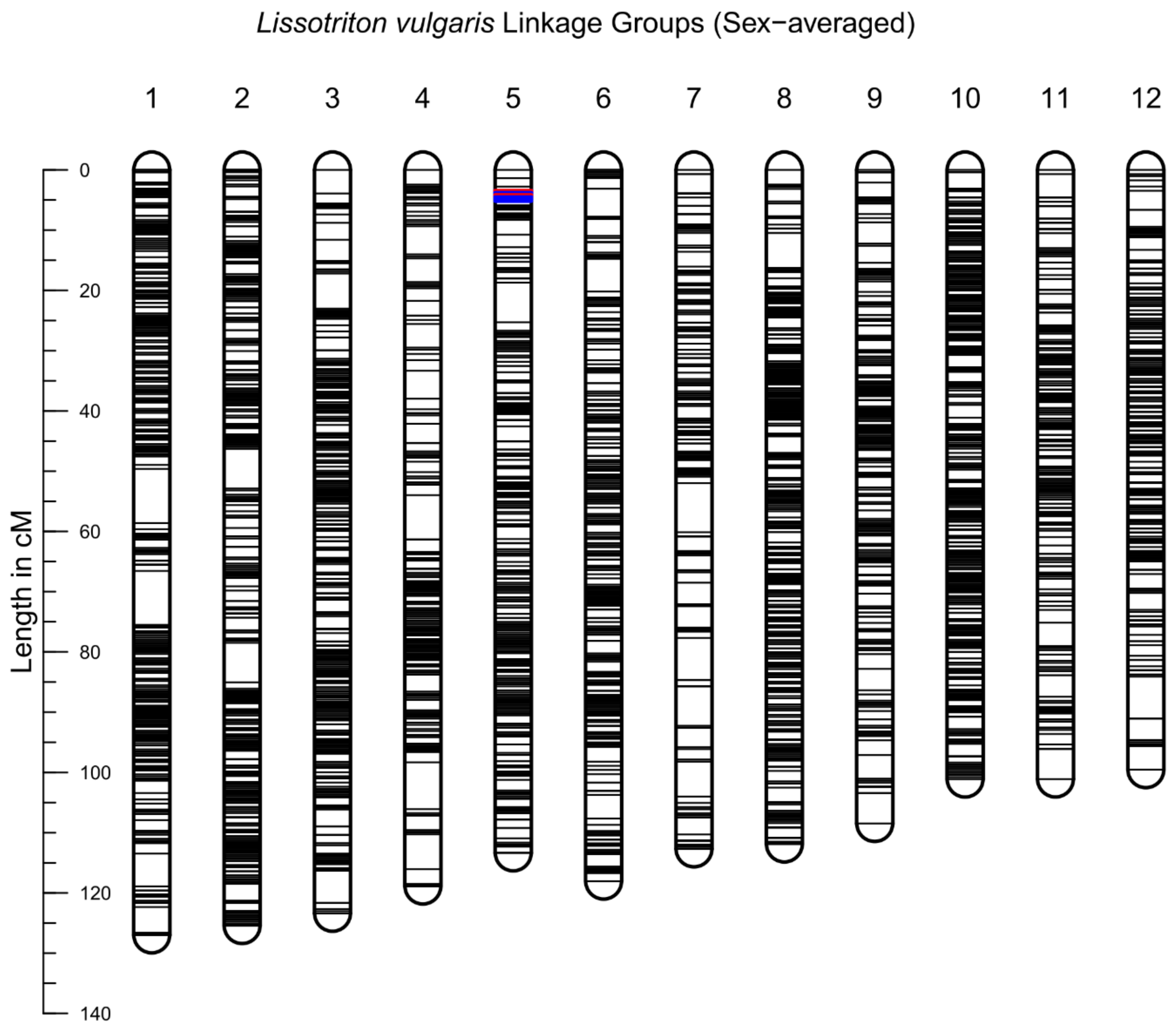

Figure S9: The sex-averaged *L. vulgaris* linkage map incorporating both the 32 Y-linked markers identified via Stacks (red) and the 24 additional Y-linked markers identified via RADsex (blue) - note that 14 markers appear in both analyses. Both sets of markers cluster tightly within the same 2 cM region of linkage group 5.

MOLECULAR ECOLOGY
RESOURCES

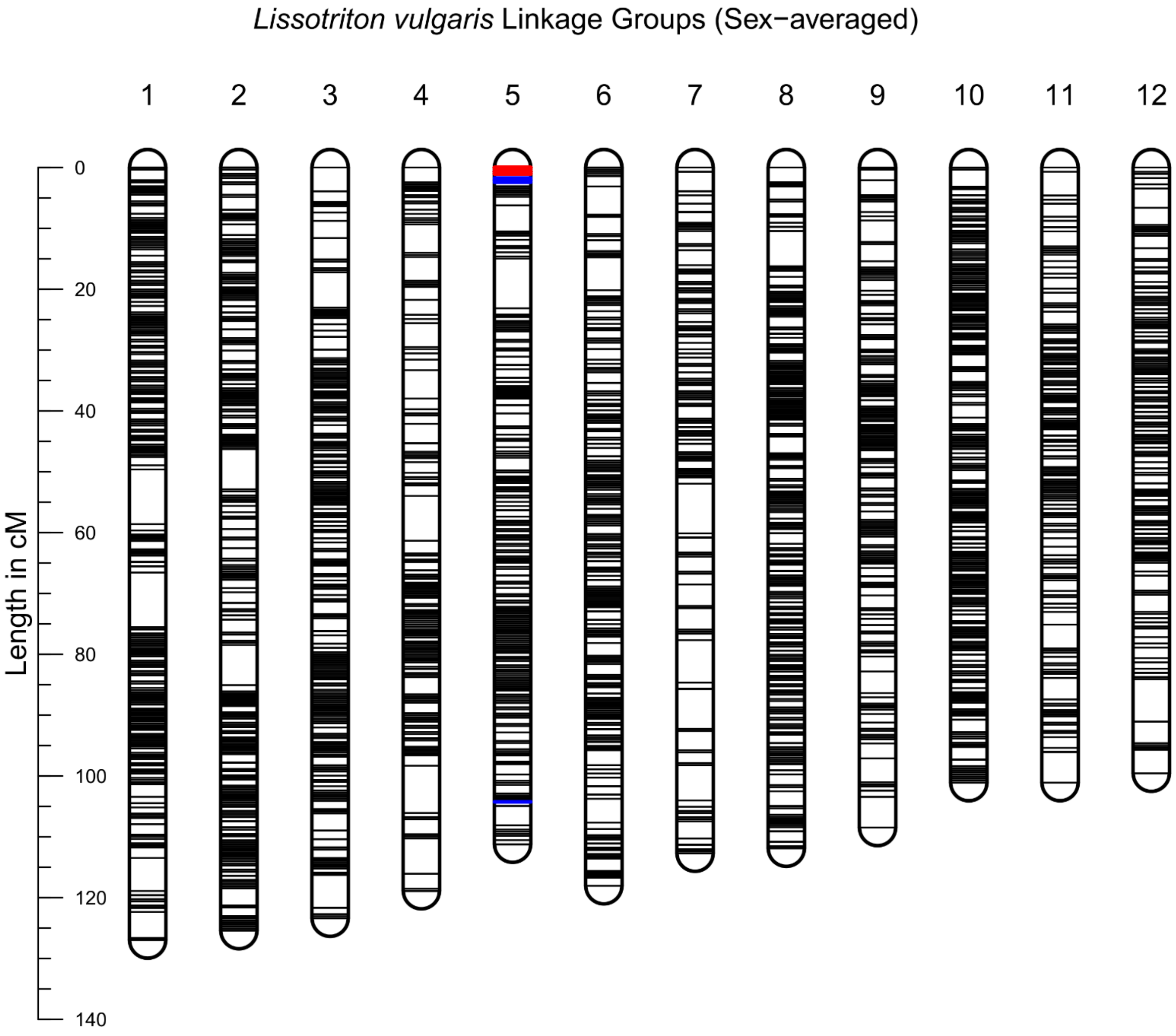

Figure S10: The sex-averaged *L. vulgaris* linkage map incorporating both the 32 Y-linked presence/absence markers identified via Stacks (red) and the eight additional X/Y-linked markers (blue) identified via incorporating data on offspring sex (by genotyping the Y-linked markers in their RADseq data) into the *ParentCall2* module of Lep-MAP 3. The additional X/Y-linked markers are all placed on linkage group 5, with all but one located less than 2 cM away from the main cluster 32 Y-linked presence/absence markers. A single additional X/Y-linked linked marker is located at the opposite end of the linkage group (likely due to an error in genotyping, or Lep-MAP 3's marker identification or ordering).

Table S1: Characteristics of the sex-averaged, paternal and maternal *Lissotriton vulgaris* linkage maps, including the number of markers and the length (in centimorgans) of each group.

Group	Sex-averaged		Paternal		Maternal	
	N markers	Length (cM)	N markers	Length (cM)	N markers	Length (cM)
**1**	1205	127.0	820	121.7	863	153.7
**2**	1317	125.4	924	126.9	916	157.0
**3**	1030	123.4	718	144.4	749	153.9
**4**	560	118.9	418	95.8	343	132.1
**5**	877	118.4	666	78.5	531	138.2
**6**	769	118.0	522	100.8	535	137.5
**7**	387	112.7	250	109.1	287	143.3
**8**	1164	111.9	831	114.1	789	151.7
**9**	918	108.5	626	85.0	648	116.7
**10**	1354	101.1	897	123.1	972	135.4
**11**	584	101.1	392	102.3	411	117.2
**12**	565	99.6	387	98.2	408	150.9

Table S2: Distribution of BLAST hits of markers from the *Lissotriton vulgaris* linkage map within the genome assembly of *Pleurodeles waltl* (Brown et al., 2025) (as seen in Fig. 3).

L. vulgaris **Linkage Group**	***P. waltl*** **Chromosome**	**Total BLAST hits**	**BLAST hits on homologous chromosome**	**% of hits on homologous chromosome**
**1**	1	61	51	83.6
**2**	4	65	56	86.2
**3**	2	52	43	82.7
**4**	11	36	31	86.1
**5**	5	44	31	70.5
**6**	8	56	43	76.8
**7**	12	21	17	81.0
**8**	6	69	56	81.2
**9**	7	56	45	80.4
**10**	3	66	53	80.3
**11**	10	25	23	92.0
**12**	9	29	24	82.8

Table S3: Sequences of all primers used in this study, CDK-17 is an autosomal marker used as a control, all others are candidate Y-linked markers developed for *Lissotriton vulgaris*.

Primer Pair	Forward Primer Sequence	Reverse Primer Sequence	Product Length (bp)
**CDK-17**	GGCATGGGAAGAACAGAAGA	CCATCTGCTTGGACTGTTGA	537
**lvY-11521-long**	GCATTTGGGCAGCTTCATTC	CAATTCAGGCACACACCAGC	>200
**lvY-28978-long**	TCATGCATAGCCAAAGAGTTTGTC	CCCTGATGACACTTGATCGC	>200
**lvY-102891-short**	CTAGATGCGCATCCACTGGG	CTGACATTAAGCAAGCCGCC	87
**lvY-102891-long**	GCGGCTTGCTTAATGTCAGG	CCCATAGTCTCCATGCCCTC	>200
**lvY-99941-long**	TTGCTGTGTGTACGTGCCAG	CGTTTGGATGGGATACAAGCAG	>200
**lvY-138925-short**	TGCCAATGACCAGCTCCTAC	TGGTAGCTACTCCTGGTGAAG	115
**lvY-138925-long**	TGCCAATGACCAGCTCCTAC	TCCACGAAGAACTGATAGAACTC	>200
**lvY-81842-short**	CTAGAATCTGCGGCGTCATG	TGAAGGTCACACTTTCCGCG	92
**lvY-81842-long**	TCAGTATGCCGTCTAGCTGC	ACCAGAGCCCCCGTTTATTG	>200
**lvY-143365-short**	TAGGGATCAGTTGGGGGAAC	CCGCAAAGCAAAAGAGACCC	106
**lvY-143365-long**	CCAGCATAAGGTGAGGAGGG	TACTGAAAAACCTGGCCCCC	>200
**lvY-51393-short**	GACCACTGTAGAGGAGGTTGG	GCTGCCTGTTTCTGGATGTC	124
**lvY-51393-long**	GACCACTGTAGAGGAGGTTGG	GATCCGTGGAGGTCGGTAAC	>200
**lvY-128014-short**	TTTTTGGGGGCTCTGCAGG	TGCTCAGTGTCTGTATCCTCTC	91
**lvY-128014-long**	GCGAGTAGATGGAAGGGTGG	TTGTTTGTCTTGCCCTTTGG	>200
**lvY-65590-short**	GCAGTGCAGTTCAGAGCATG	AGCCAGCACAAACAGATAGAG	104
**lvY-65590-long**	GCAGTGCAGTTCAGAGCATG	CAAAGCCTGTGTGCCAACTC	>200
**lvY-36220-short**	CTAGACTCACGCACACACCC	CCTCCTCCTCTCTCCCTAGC	97
**lvY-36220-long**	ACTGGTGCTAGGGAGAGAGG	GGCTTTCTTTCTCAGCACAGC	>200
**lvY-123701-short**	AGGCCTCAGTTCTTCTTGGG	GGTCCACTGTCCACATTGTG	126
**lvY-123701-long**	TGTTGCATTAGTCCTCTCCCC	GCAATTACGGACTCAGCGTTC	>200
**lvY-115632-short**	ACTCTACTGATACTTGCCATGCC	TGTCATCGAGCTTAGGCCAC	95
**lvY-115632-long**	TGTGGCCTAAGCTCGATGAC	ATTCCTCAGGGCTGTTGCAG	>200
**lvY-79267-short**	CAAGGCCAAAATGATCCCGC	ACTCTGGGAGCAGTAGTCAC	107
**lvY-79267-long**	CAAGGCCAAAATGATCCCGC	TGTGCATTGACCATAAAGCCC	>200

MOLECULAR ECOLOGY RESOURCES

Table S4: Details of all *Lissotriton* samples used in this study. Samples denoted with * are also reported in Babik et al. (2024). All samples for which RADseq was performed are available via NCBI, associated with Bioproject: PRJNA1118769 (France et al., 2024).

Sample	Species	Sex	Used for	Country	Location	Latitude	Longitude	SRA accession
**1463**	*L. vulgaris ampelensis*	F	Screening	Romania	Arieseni	46.465	22.783	NA
**2568**	*L. italicus*	M	Screening	Italy	Conversano	40.97	17.12	NA
**2575**	*L. boscai*	M	Screening	Spain	Pelahustán	40.17	-4.59	NA
**2612**	*L. vulgaris*	M	Screening, Multiplex validation	Poland	Maćkowice	49.857	22.716	NA
**2613**	*L. vulgaris*	F	Screening, Multiplex validation	Poland	Maćkowice	49.857	22.716	NA
**2976**	*L. montandoni*	F	Screening, Multiplex validation	Poland	Lachowice	49.683	19.419	NA
**3011**	*L. montandoni*	M	Screening, Multiplex validation	Poland	Smerek	49.162	22.436	NA
**3995**	*L. graecus*	F	Screening	Greece	Girtoni	39.736	22.47	NA
**4333**	*L. vulgaris meridionalis*	F	Screening	Croatia	Salakovac	45.053	14.087	NA
**4343**	*L. vulgaris meridionalis*	M	Screening	Croatia	Salakovac	45.053	14.087	NA
**4991**	*L. schmidtleri*	M	Screening	Turkey	Gulluce	40.071	28.353	NA
**5511**	*L. schmidtleri*	F	Screening	Bulgaria	Lozenets	42.206	27.802	NA
**6358**	*L. kosswigi*	F	Screening	Turkey	Mollafeneri	40.893	29.507	NA
**6386**	*L. kosswigi*	M	Screening	Turkey	Denizli	40.893	29.564	NA
**7533**	*L. vulgaris ampelensis*	M	Screening	Romania	Remetea	46.809	25.372	NA
**8419**	*L. helveticus*	M	Screening	France	Jublain	48.234	-0.527	NA
**10479**	*L. graecus*	M	Screening	Montenegro	Razvuđska Kamenica	42.692	18.723	NA
**12800**	*L. helveticus*	F	Screening	Spain	La Manjoya	43.334	-5.868	NA
**12840**	*L. boscai*	F	Screening	Spain	Lamuño	43.563	-6.2	NA
**13656**	*L. italicus*	F	Screening	Italy	Gorga	41.648	13.07	NA
**2185**	*L. vulgaris*	F	Validation, Multiplex validation	Poland	Pokrzywna	50.293	17.454	NA
**2189**	*L. vulgaris*	F	Validation, Multiplex validation	Poland	Pokrzywna	50.293	17.454	NA
**2526**	*L. vulgaris*	M	Validation, Multiplex validation	Poland	Brończyce	50.287	20.528	NA
**2584**	*L. vulgaris*	M	Validation, Multiplex validation	Poland	Kuźmina	49.619	22.428	NA
**2587**	*L. vulgaris*	M	Validation, Multiplex validation	Poland	Kuźmina	49.619	22.428	NA
**2598**	*L. vulgaris*	F	Validation, Multiplex validation	Poland	Kuźmina	49.619	22.428	NA

**2599**	*L. vulgaris*	F	Validation, Multiplex validation	Poland	Kuźmina	49.619	22.428	NA
**2600**	*L. vulgaris*	F	Validation, Multiplex validation	Poland	Kuźmina	49.619	22.428	NA
**2602**	*L. vulgaris*	M	Validation, Multiplex validation	Poland	Maćkowice	49.857	22.716	NA
**2603**	*L. vulgaris*	M	Validation, Multiplex validation	Poland	Maćkowice	49.857	22.716	NA
**2605**	*L. vulgaris*	M	Validation, Multiplex validation	Poland	Maćkowice	49.857	22.716	NA
**2607**	*L. vulgaris*	F	Validation, Multiplex validation	Poland	Maćkowice	49.857	22.716	NA
**2609**	*L. vulgaris*	F	Multiplex validation	Poland	Maćkowice	49.857	22.716	NA
**2610**	*L. vulgaris*	F	Multiplex validation	Poland	Maćkowice	49.857	22.716	NA
**2614**	*L. vulgaris*	F	Multiplex validation	Poland	Maćkowice	49.857	22.716	NA
**2615**	*L. vulgaris*	F	Multiplex validation	Poland	Maćkowice	49.857	22.716	NA
**3762**	*L. vulgaris*	F	Multiplex validation	Poland	Kraków	50.061	19.920	NA
**10764**	*L. vulgaris*	F	Multiplex validation	Poland	Szymbark	49.604	21.088	NA
**14966**	*L. vulgaris*	F	Multiplex validation	Poland	Iskań	49.790	22.441	NA
**14967**	*L. vulgaris*	F	Multiplex validation	Poland	Iskań	49.790	22.441	NA
**14968**	*L. vulgaris*	F	Multiplex validation	Poland	Iskań	49.790	22.441	NA
**14969**	*L. vulgaris*	F	Multiplex validation	Poland	Iskań	49.790	22.441	NA
**14976**	*L. vulgaris*	F	Multiplex validation	Poland	Iskań	49.790	22.441	NA
**15005**	*L. vulgaris*	F	Multiplex validation	Poland	Malawa	49.692	22.425	NA
**15006**	*L. vulgaris*	F	Multiplex validation	Poland	Malawa	49.692	22.425	NA
**2612**	*L. vulgaris*	M	Multiplex validation	Poland	Maćkowice	49.857	22.716	NA
**2620**	*L. vulgaris*	M	Multiplex validation	Poland	Jasło	49.739	21.493	NA
**3090**	*L. vulgaris*	M	Multiplex validation	Poland	Samostrzałów	50.592	20.641	NA
**3091**	*L. vulgaris*	M	Multiplex validation	Poland	Samostrzałów	50.592	20.641	NA
**10763**	*L. vulgaris*	M	Multiplex validation	Poland	Dziewin	50.080	20.405	NA
**13636**	*L. vulgaris*	M	Multiplex validation	Poland	Kuźmina	49.619	22.428	NA
**14963**	*L. vulgaris*	M	Multiplex validation	Poland	Iskań	49.790	22.441	NA
**14964**	*L. vulgaris*	M	Multiplex validation	Poland	Iskań	49.790	22.441	NA
**14965**	*L. vulgaris*	M	Multiplex validation	Poland	Iskań	49.790	22.441	NA
**14975**	*L. vulgaris*	M	Multiplex validation	Poland	Iskań	49.790	22.441	NA
**14996**	*L. vulgaris*	M	Multiplex validation	Poland	Malawa	49.692	22.425	NA

**14998**	*L. vulgaris*	M	Multiplex validation	Poland	Malawa	49.692	22.425	NA
**14999**	*L. vulgaris*	M	Multiplex validation	Poland	Malawa	49.692	22.425	NA
**15000**	*L. vulgaris*	M	Multiplex validation	Poland	Malawa	49.692	22.425	NA
**15002**	*L. vulgaris*	M	Multiplex validation	Poland	Malawa	49.692	22.425	NA
**15003**	*L. vulgaris*	M	Multiplex validation	Poland	Malawa	49.692	22.425	NA
**1843**	*L. montandoni*	F	Multiplex validation	Poland	Smerek	49.162	22.436	NA
**2586**	*L. montandoni*	F	Multiplex validation	Poland	Kuźmina	49.619	22.428	NA
**2593**	*L. montandoni*	F	Multiplex validation	Poland	Kuźmina	49.619	22.428	NA
**2975**	*L. montandoni*	F	Multiplex validation	Poland	Lachowice	49.683	19.419	NA
**2977**	*L. montandoni*	F	Multiplex validation	Poland	Lachowice	49.683	19.419	NA
**2979**	*L. montandoni*	F	Multiplex validation	Poland	Lachowice	49.683	19.419	NA
**2980**	*L. montandoni*	F	Multiplex validation	Poland	Lachowice	49.683	19.419	NA
**2981**	*L. montandoni*	F	Multiplex validation	Poland	Lachowice	49.683	19.419	NA
**2983**	*L. montandoni*	F	Multiplex validation	Poland	Lachowice	49.683	19.419	NA
**9306**	*L. montandoni*	F	Multiplex validation	Ukraine	Turje	49.286	23.112	NA
**9307**	*L. montandoni*	F	Multiplex validation	Ukraine	Turje	49.286	23.112	NA
**9308**	*L. montandoni*	F	Multiplex validation	Ukraine	Turje	49.286	23.112	NA
**9309**	*L. montandoni*	F	Multiplex validation	Ukraine	Turje	49.286	23.112	NA
**9310**	*L. montandoni*	F	Multiplex validation	Ukraine	Turje	49.286	23.112	NA
**9311**	*L. montandoni*	F	Multiplex validation	Ukraine	Turje	49.286	23.112	NA
**9312**	*L. montandoni*	F	Multiplex validation	Ukraine	Turje	49.286	23.112	NA
**9313**	*L. montandoni*	F	Multiplex validation	Ukraine	Turje	49.286	23.112	NA
**9314**	*L. montandoni*	F	Multiplex validation	Ukraine	Turje	49.286	23.112	NA
**9315**	*L. montandoni*	F	Multiplex validation	Ukraine	Turje	49.286	23.112	NA
**9316**	*L. montandoni*	F	Multiplex validation	Ukraine	Turje	49.286	23.112	NA
**9317**	*L. montandoni*	F	Multiplex validation	Ukraine	Turje	49.286	23.112	NA
**14962**	*L. montandoni*	F	Multiplex validation	Poland	Iskań	49.790	22.441	NA
**3010**	*L. montandoni*	M	Multiplex validation	Poland	Smerek	49.162	22.436	NA
**9300**	*L. montandoni*	M	Multiplex validation	Ukraine	Turje	49.286	23.112	NA
**9301**	*L. montandoni*	M	Multiplex validation	Ukraine	Turje	49.286	23.112	NA
**9302**	*L. montandoni*	M	Multiplex validation	Ukraine	Turje	49.286	23.112	NA

**9303**	*L. montandoni*	M	Multiplex validation	Ukraine	Turje	49.286	23.112	NA
**9304**	*L. montandoni*	M	Multiplex validation	Ukraine	Turje	49.286	23.112	NA
**9305**	*L. montandoni*	M	Multiplex validation	Ukraine	Turje	49.286	23.112	NA
**13635**	*L. montandoni*	M	Multiplex validation	Poland	Kuźmina	49.619	22.428	NA
**14950**	*L. montandoni*	M	Multiplex validation	Poland	Iskań	49.790	22.441	NA
**14951**	*L. montandoni*	M	Multiplex validation	Poland	Iskań	49.790	22.441	NA
**14954**	*L. montandoni*	M	Multiplex validation	Poland	Iskań	49.790	22.441	NA
**14957**	*L. montandoni*	M	Multiplex validation	Poland	Iskań	49.790	22.441	NA
**14958**	*L. montandoni*	M	Multiplex validation	Poland	Iskań	49.790	22.441	NA
**14959**	*L. montandoni*	M	Multiplex validation	Poland	Iskań	49.790	22.441	NA
**14970**	*L. montandoni*	M	Multiplex validation	Poland	Iskań	49.790	22.441	NA
**14971**	*L. montandoni*	M	Multiplex validation	Poland	Iskań	49.790	22.441	NA
**14972**	*L. montandoni*	M	Multiplex validation	Poland	Iskań	49.790	22.441	NA
**14973**	*L. montandoni*	M	Multiplex validation	Poland	Iskań	49.790	22.441	NA
**14974**	*L. montandoni*	M	Multiplex validation	Poland	Iskań	49.790	22.441	NA
**14977**	*L. montandoni*	M	Multiplex validation	Poland	Malawa	49.690	22.415	NA
**14979**	*L. montandoni*	M	Multiplex validation	Poland	Malawa	49.690	22.415	NA
**14980**	*L. montandoni*	M	Multiplex validation	Poland	Malawa	49.690	22.415	NA
**14981**	*L. montandoni*	M	Multiplex validation	Poland	Malawa	49.690	22.415	NA
**18451***	*L. vulgaris*	M	Known-sex RADseq	Poland	Kraków Dziewin	50.0757	20.4111	SRR29248882
**18487***	*L. vulgaris*	M	Known-sex RADseq	Poland	Kraków Botanical Garden	50.0624	19.9571	SRR29248881
**18488***	*L. vulgaris*	M	Known-sex RADseq	Poland	Kraków Botanical Garden	50.0624	19.9571	SRR29248694
**18489***	*L. vulgaris*	F	Known-sex RADseq	Poland	Kraków Botanical Garden	50.0624	19.9571	SRR29248675
**18490***	*L. vulgaris*	F	Known-sex RADseq	Poland	Kraków Botanical Garden	50.0624	19.9571	SRR29248772
**18491***	*L. vulgaris*	F	Known-sex RADseq	Poland	Kraków Botanical Garden	50.0624	19.9571	SRR29248761
**18492***	*L. vulgaris*	F	Known-sex RADseq	Poland	Kraków Botanical Garden	50.0624	19.9571	SRR29248846
**18493***	*L. vulgaris*	F	Known-sex RADseq	Poland	Kraków Botanical Garden	50.0624	19.9571	SRR29248835
**18494***	*L. vulgaris*	F	Known-sex RADseq	Poland	Kraków Botanical Garden	50.0624	19.9571	SRR29248824
**18495***	*L. vulgaris*	F	Known-sex RADseq	Poland	Kraków Botanical Garden	50.0624	19.9571	SRR29248717
**18496***	*L. vulgaris*	F	Known-sex RADseq	Poland	Kraków Botanical Garden	50.0624	19.9571	SRR29248880

**18497***	*L. vulgaris*	F	Known-sex RADseq	Poland	Kraków Botanical Garden	50.0624	19.9571	SRR29248805
**18498***	*L. vulgaris*	F	Known-sex RADseq	Poland	Kraków Botanical Garden	50.0624	19.9571	SRR29248794
**18499***	*L. vulgaris*	F	Known-sex RADseq	Poland	Kraków Botanical Garden	50.0624	19.9571	SRR29248783
**18500***	*L. vulgaris*	F	Known-sex RADseq	Poland	Kraków Botanical Garden	50.0624	19.9571	SRR29248868
**18501***	*L. vulgaris*	F	Known-sex RADseq	Poland	Kraków Botanical Garden	50.0624	19.9571	SRR29248857
**18502***	*L. vulgaris*	F	Known-sex RADseq	Poland	Kraków Botanical Garden	50.0624	19.9571	SRR29248750
**19348***	*L. vulgaris*	M	Known-sex RADseq	Poland	Kraków Park Wenedy	50.0211	20.0019	SRR29248739
**19349***	*L. vulgaris*	M	Known-sex RADseq	Poland	Kraków Park Wenedy	50.0211	20.0019	SRR29248728
**19376***	*L. vulgaris*	M	Known-sex RADseq	Poland	Kraków Słona Woda	50.006	19.9963	SRR29248705
**19424***	*L. vulgaris*	F	Known-sex RADseq	Poland	Kraków Królówka	50.0253	19.8673	SRR29248693
**19427***	*L. vulgaris*	F	Known-sex RADseq	Poland	Kraków Królówka	50.0253	19.8673	SRR29248684
**19430***	*L. vulgaris*	M	Known-sex RADseq	Poland	Kraków Wzgórze Grodzisko	50.0047	19.7993	SRR29248683
**19431***	*L. vulgaris*	M	Known-sex RADseq	Poland	Kraków Wzgórze Grodzisko	50.0047	19.7993	SRR29248682
**19433***	*L. vulgaris*	F	Known-sex RADseq	Poland	Kraków Wzgórze Grodzisko	50.0047	19.7993	SRR29248681
**19434***	*L. vulgaris*	F	Known-sex RADseq	Poland	Kraków Wzgórze Grodzisko	50.0047	19.7993	SRR29248680
**19435***	*L. vulgaris*	M	Known-sex RADseq	Poland	Kraków Wzgórze Grodzisko	50.0047	19.7993	SRR29248679
**19437***	*L. vulgaris*	M	Known-sex RADseq	Poland	Kraków Wzgórze Grodzisko	50.0047	19.7993	SRR29248678
**19439***	*L. vulgaris*	M	Known-sex RADseq	Poland	Kraków Wzgórze Grodzisko	50.0047	19.7993	SRR29248677
**19649***	*L. vulgaris*	M	Known-sex RADseq	Poland	Kraków Liban	50.0372	19.9567	SRR29248676
**19651***	*L. vulgaris*	F	Known-sex RADseq	Poland	Kraków Liban	50.0372	19.9567	SRR29248782
**19652***	*L. vulgaris*	M	Known-sex RADseq	Poland	Kraków Liban	50.0372	19.9567	SRR29248781
**19653***	*L. vulgaris*	F	Known-sex RADseq	Poland	Kraków Liban	50.0372	19.9567	SRR29248780
**19655***	*L. vulgaris*	M	Known-sex RADseq	Poland	Kraków Liban	50.0372	19.9567	SRR29248779
**19658***	*L. vulgaris*	F	Known-sex RADseq	Poland	Kraków Liban	50.0372	19.9567	SRR29248778
**19660***	*L. vulgaris*	M	Known-sex RADseq	Poland	Kraków Liban	50.0372	19.9567	SRR29248777
**19666***	*L. vulgaris*	M	Known-sex RADseq	Poland	Kraków Liban	50.0372	19.9567	SRR29248776
**19667***	*L. vulgaris*	M	Known-sex RADseq	Poland	Kraków Niedźwiedzia Góra	50.1017	19.6243	SRR29248775
**19669***	*L. vulgaris*	M	Known-sex RADseq	Poland	Kraków Niedźwiedzia Góra	50.1017	19.6243	SRR29248774
**19672***	*L. vulgaris*	F	Known-sex RADseq	Poland	Kraków Niedźwiedzia Góra	50.1024	19.6239	SRR29248773
**19673***	*L. vulgaris*	F	Known-sex RADseq	Poland	Kraków Niedźwiedzia Góra	50.1024	19.6239	SRR29248771

MOLECULAR ECOLOGY RESOURCES

**19674***	*L. vulgaris*	M	Known-sex RADseq	Poland	Kraków Niedźwiedzia Góra	50.1024	19.6239	SRR29248770
**19675***	*L. vulgaris*	F	Known-sex RADseq	Poland	Kraków Niedźwiedzia Góra	50.1024	19.6239	SRR29248769
**19676***	*L. vulgaris*	F	Known-sex RADseq	Poland	Kraków Niedźwiedzia Góra	50.1024	19.6239	SRR29248768
**19677***	*L. vulgaris*	M	Known-sex RADseq	Poland	Kraków Niedźwiedzia Góra	50.1024	19.6239	SRR29248767
**19679***	*L. vulgaris*	F	Known-sex RADseq	Poland	Kraków Niedźwiedzia Góra	50.1024	19.6239	SRR29248766
**19682***	*L. vulgaris*	F	Known-sex RADseq	Poland	Kraków Niedźwiedzia Góra	50.1024	19.6239	SRR29248765
**23036***	*L. vulgaris*	F	Known-sex RADseq	Poland	Brończyce	50.2314	20.1193	SRR29248764
**23038***	*L. vulgaris*	F	Known-sex RADseq	Poland	Kraków Proszowice	50.1979	20.2828	SRR29248763
**23039***	*L. vulgaris*	F	Known-sex RADseq	Poland	Kraków Proszowice	50.1979	20.2828	SRR29248762
**23043***	*L. vulgaris*	M	Known-sex RADseq	Poland	Kraków Proszowice	50.1979	20.2828	SRR29248760
**23044***	*L. vulgaris*	M	Known-sex RADseq	Poland	Kraków Proszowice	50.1979	20.2828	SRR29248759
**23045***	*L. vulgaris*	M	Known-sex RADseq	Poland	Kraków Proszowice	50.1979	20.2828	SRR29248758
**23046***	*L. vulgaris*	M	Known-sex RADseq	Poland	Kraków Proszowice	50.1979	20.2828	SRR29248757
**23047***	*L. vulgaris*	M	Known-sex RADseq	Poland	Kraków Proszowice	50.1979	20.2828	SRR29248756
**23048***	*L. vulgaris*	M	Known-sex RADseq	Poland	Kraków Proszowice	50.1979	20.2828	SRR29248755
**23051***	*L. vulgaris*	M	Known-sex RADseq	Poland	Kraków Proszowice	50.1979	20.2828	SRR29248754
**23054***	*L. vulgaris*	M	Known-sex RADseq	Poland	Brończyce	50.2314	20.1193	SRR29248753
**23058***	*L. vulgaris*	M	Known-sex RADseq	Poland	Brończyce	50.2314	20.1193	SRR29248752
**23064***	*L. vulgaris*	M	Known-sex RADseq	Poland	Kraków Niedźwiedzia Góra	50.1026	19.6237	SRR29248751
**17011**	*L. vulgaris*	F	Linkage map parent, Multiplex validation	Poland	Kraków Zakrzówek Park	50.04	19.9168	SRR29248845
**17012**	*L. vulgaris*	M	Linkage map parent, Multiplex validation	Poland	Kraków Zakrzówek Park	50.04	19.9168	SRR29248844
**20856**	*L. vulgaris*	Unknown	Linkage map progeny	NA	NA	NA	NA	SRR29248843
**20857**	*L. vulgaris*	Unknown	Linkage map progeny	NA	NA	NA	NA	SRR29248842
**20858**	*L. vulgaris*	Unknown	Linkage map progeny	NA	NA	NA	NA	SRR29248841
**20859**	*L. vulgaris*	Unknown	Linkage map progeny	NA	NA	NA	NA	SRR29248840
**20868**	*L. vulgaris*	Unknown	Linkage map progeny	NA	NA	NA	NA	SRR29248839
**20870**	*L. vulgaris*	Unknown	Linkage map progeny	NA	NA	NA	NA	SRR29248838
**20871**	*L. vulgaris*	Unknown	Linkage map progeny	NA	NA	NA	NA	SRR29248837
**20872**	*L. vulgaris*	Unknown	Linkage map progeny	NA	NA	NA	NA	SRR29248836
**20873**	*L. vulgaris*	Unknown	Linkage map progeny	NA	NA	NA	NA	SRR29248834

**20875**	*L. vulgaris*	Unknown	Linkage map progeny	NA	NA	NA	NA	SRR29248833
**20876**	*L. vulgaris*	Unknown	Linkage map progeny	NA	NA	NA	NA	SRR29248832
**20877**	*L. vulgaris*	Unknown	Linkage map progeny	NA	NA	NA	NA	SRR29248831
**20878**	*L. vulgaris*	Unknown	Linkage map progeny	NA	NA	NA	NA	SRR29248830
**20880**	*L. vulgaris*	Unknown	Linkage map progeny	NA	NA	NA	NA	SRR29248829
**20881**	*L. vulgaris*	Unknown	Linkage map progeny	NA	NA	NA	NA	SRR29248828
**20883**	*L. vulgaris*	Unknown	Linkage map progeny	NA	NA	NA	NA	SRR29248827
**20884**	*L. vulgaris*	Unknown	Linkage map progeny	NA	NA	NA	NA	SRR29248826
**20885**	*L. vulgaris*	Unknown	Linkage map progeny	NA	NA	NA	NA	SRR29248825
**20887**	*L. vulgaris*	Unknown	Linkage map progeny	NA	NA	NA	NA	SRR29248823
**20888**	*L. vulgaris*	Unknown	Linkage map progeny	NA	NA	NA	NA	SRR29248822
**20890**	*L. vulgaris*	Unknown	Linkage map progeny	NA	NA	NA	NA	SRR29248821
**20891**	*L. vulgaris*	Unknown	Linkage map progeny	NA	NA	NA	NA	SRR29248820
**20892**	*L. vulgaris*	Unknown	Linkage map progeny	NA	NA	NA	NA	SRR29248819
**20894**	*L. vulgaris*	Unknown	Linkage map progeny	NA	NA	NA	NA	SRR29248818
**20895**	*L. vulgaris*	Unknown	Linkage map progeny	NA	NA	NA	NA	SRR29248817
**20896**	*L. vulgaris*	Unknown	Linkage map progeny	NA	NA	NA	NA	SRR29248816
**20897**	*L. vulgaris*	Unknown	Linkage map progeny	NA	NA	NA	NA	SRR29248815
**20901**	*L. vulgaris*	Unknown	Linkage map progeny	NA	NA	NA	NA	SRR29248718
**20903**	*L. vulgaris*	Unknown	Linkage map progeny	NA	NA	NA	NA	SRR29248716
**20905**	*L. vulgaris*	Unknown	Linkage map progeny	NA	NA	NA	NA	SRR29248715
**20906**	*L. vulgaris*	Unknown	Linkage map progeny	NA	NA	NA	NA	SRR29248714
**20908**	*L. vulgaris*	Unknown	Linkage map progeny	NA	NA	NA	NA	SRR29248713
**20909**	*L. vulgaris*	Unknown	Linkage map progeny	NA	NA	NA	NA	SRR29248712
**20910**	*L. vulgaris*	Unknown	Linkage map progeny	NA	NA	NA	NA	SRR29248711
**20911**	*L. vulgaris*	Unknown	Linkage map progeny	NA	NA	NA	NA	SRR29248710
**20912**	*L. vulgaris*	Unknown	Linkage map progeny	NA	NA	NA	NA	SRR29248709
**20914**	*L. vulgaris*	Unknown	Linkage map progeny	NA	NA	NA	NA	SRR29248708
**20915**	*L. vulgaris*	Unknown	Linkage map progeny	NA	NA	NA	NA	SRR29248707
**20916**	*L. vulgaris*	Unknown	Linkage map progeny	NA	NA	NA	NA	SRR29248879

**20917**	*L. vulgaris*	Unknown	Linkage map progeny	NA	NA	NA	NA	SRR29248814
**20918**	*L. vulgaris*	Unknown	Linkage map progeny	NA	NA	NA	NA	SRR29248813
**20919**	*L. vulgaris*	Unknown	Linkage map progeny	NA	NA	NA	NA	SRR29248812
**20920**	*L. vulgaris*	Unknown	Linkage map progeny	NA	NA	NA	NA	SRR29248811
**20921**	*L. vulgaris*	Unknown	Linkage map progeny	NA	NA	NA	NA	SRR29248810
**20922**	*L. vulgaris*	Unknown	Linkage map progeny	NA	NA	NA	NA	SRR29248809
**20925**	*L. vulgaris*	Unknown	Linkage map progeny	NA	NA	NA	NA	SRR29248808
**20926**	*L. vulgaris*	Unknown	Linkage map progeny	NA	NA	NA	NA	SRR29248807
**20927**	*L. vulgaris*	Unknown	Linkage map progeny	NA	NA	NA	NA	SRR29248806
**20928**	*L. vulgaris*	Unknown	Linkage map progeny	NA	NA	NA	NA	SRR29248804
**20929**	*L. vulgaris*	Unknown	Linkage map progeny	NA	NA	NA	NA	SRR29248803
**20930**	*L. vulgaris*	Unknown	Linkage map progeny	NA	NA	NA	NA	SRR29248802
**20932**	*L. vulgaris*	Unknown	Linkage map progeny	NA	NA	NA	NA	SRR29248801
**20936**	*L. vulgaris*	Unknown	Linkage map progeny	NA	NA	NA	NA	SRR29248800
**20938**	*L. vulgaris*	Unknown	Linkage map progeny	NA	NA	NA	NA	SRR29248799
**20939**	*L. vulgaris*	Unknown	Linkage map progeny	NA	NA	NA	NA	SRR29248798
**20941**	*L. vulgaris*	Unknown	Linkage map progeny	NA	NA	NA	NA	SRR29248797
**20942**	*L. vulgaris*	Unknown	Linkage map progeny	NA	NA	NA	NA	SRR29248796
**20943**	*L. vulgaris*	Unknown	Linkage map progeny	NA	NA	NA	NA	SRR29248795
**20944**	*L. vulgaris*	Unknown	Linkage map progeny	NA	NA	NA	NA	SRR29248793
**20945**	*L. vulgaris*	Unknown	Linkage map progeny	NA	NA	NA	NA	SRR29248792
**20946**	*L. vulgaris*	Unknown	Linkage map progeny	NA	NA	NA	NA	SRR29248791
**20947**	*L. vulgaris*	Unknown	Linkage map progeny	NA	NA	NA	NA	SRR29248790
**20949**	*L. vulgaris*	Unknown	Linkage map progeny	NA	NA	NA	NA	SRR29248789
**20950**	*L. vulgaris*	Unknown	Linkage map progeny	NA	NA	NA	NA	SRR29248788
**20951**	*L. vulgaris*	Unknown	Linkage map progeny	NA	NA	NA	NA	SRR29248787
**20952**	*L. vulgaris*	Unknown	Linkage map progeny	NA	NA	NA	NA	SRR29248786
**20953**	*L. vulgaris*	Unknown	Linkage map progeny	NA	NA	NA	NA	SRR29248785
**20956**	*L. vulgaris*	Unknown	Linkage map progeny	NA	NA	NA	NA	SRR29248784
**20957**	*L. vulgaris*	Unknown	Linkage map progeny	NA	NA	NA	NA	SRR29248878

**21060**	*L. vulgaris*	Unknown	Linkage map progeny	NA	NA	NA	NA	SRR29248877
**21061**	*L. vulgaris*	Unknown	Linkage map progeny	NA	NA	NA	NA	SRR29248876
**21062**	*L. vulgaris*	Unknown	Linkage map progeny	NA	NA	NA	NA	SRR29248875
**21065**	*L. vulgaris*	Unknown	Linkage map progeny	NA	NA	NA	NA	SRR29248874
**21066**	*L. vulgaris*	Unknown	Linkage map progeny	NA	NA	NA	NA	SRR29248873
**21067**	*L. vulgaris*	Unknown	Linkage map progeny	NA	NA	NA	NA	SRR29248872
**21068**	*L. vulgaris*	Unknown	Linkage map progeny	NA	NA	NA	NA	SRR29248871
**21069**	*L. vulgaris*	Unknown	Linkage map progeny	NA	NA	NA	NA	SRR29248870
**21070**	*L. vulgaris*	Unknown	Linkage map progeny	NA	NA	NA	NA	SRR29248869
**21071**	*L. vulgaris*	Unknown	Linkage map progeny	NA	NA	NA	NA	SRR29248867
**21072**	*L. vulgaris*	Unknown	Linkage map progeny	NA	NA	NA	NA	SRR29248866
**21073**	*L. vulgaris*	Unknown	Linkage map progeny	NA	NA	NA	NA	SRR29248865
**21074**	*L. vulgaris*	Unknown	Linkage map progeny	NA	NA	NA	NA	SRR29248864
**21080**	*L. vulgaris*	Unknown	Linkage map progeny	NA	NA	NA	NA	SRR29248863
**21081**	*L. vulgaris*	Unknown	Linkage map progeny	NA	NA	NA	NA	SRR29248862
**21083**	*L. vulgaris*	Unknown	Linkage map progeny	NA	NA	NA	NA	SRR29248861
**21084**	*L. vulgaris*	Unknown	Linkage map progeny	NA	NA	NA	NA	SRR29248860
**21086**	*L. vulgaris*	Unknown	Linkage map progeny	NA	NA	NA	NA	SRR29248859
**21087**	*L. vulgaris*	Unknown	Linkage map progeny	NA	NA	NA	NA	SRR29248858
**21088**	*L. vulgaris*	Unknown	Linkage map progeny	NA	NA	NA	NA	SRR29248856
**21089**	*L. vulgaris*	Unknown	Linkage map progeny	NA	NA	NA	NA	SRR29248855
**21090**	*L. vulgaris*	Unknown	Linkage map progeny	NA	NA	NA	NA	SRR29248854
**21094**	*L. vulgaris*	Unknown	Linkage map progeny	NA	NA	NA	NA	SRR29248853
**21095**	*L. vulgaris*	Unknown	Linkage map progeny	NA	NA	NA	NA	SRR29248852
**21098**	*L. vulgaris*	Unknown	Linkage map progeny	NA	NA	NA	NA	SRR29248851
**21100**	*L. vulgaris*	Unknown	Linkage map progeny	NA	NA	NA	NA	SRR29248850
**21101**	*L. vulgaris*	Unknown	Linkage map progeny	NA	NA	NA	NA	SRR29248849
**21102**	*L. vulgaris*	Unknown	Linkage map progeny	NA	NA	NA	NA	SRR29248848
**21103**	*L. vulgaris*	Unknown	Linkage map progeny	NA	NA	NA	NA	SRR29248847
**21104**	*L. vulgaris*	Unknown	Linkage map progeny	NA	NA	NA	NA	SRR29248749

**21105**	*L. vulgaris*	Unknown	Linkage map progeny	NA	NA	NA	NA	SRR29248748
**21106**	*L. vulgaris*	Unknown	Linkage map progeny	NA	NA	NA	NA	SRR29248747
**21108**	*L. vulgaris*	Unknown	Linkage map progeny	NA	NA	NA	NA	SRR29248746
**21111**	*L. vulgaris*	Unknown	Linkage map progeny	NA	NA	NA	NA	SRR29248745
**21117**	*L. vulgaris*	Unknown	Linkage map progeny	NA	NA	NA	NA	SRR29248744
**21118**	*L. vulgaris*	Unknown	Linkage map progeny	NA	NA	NA	NA	SRR29248743
**21119**	*L. vulgaris*	Unknown	Linkage map progeny	NA	NA	NA	NA	SRR29248742
**21120**	*L. vulgaris*	Unknown	Linkage map progeny	NA	NA	NA	NA	SRR29248741
**21121**	*L. vulgaris*	Unknown	Linkage map progeny	NA	NA	NA	NA	SRR29248740
**21123**	*L. vulgaris*	Unknown	Linkage map progeny	NA	NA	NA	NA	SRR29248738
**21124**	*L. vulgaris*	Unknown	Linkage map progeny	NA	NA	NA	NA	SRR29248737
**21125**	*L. vulgaris*	Unknown	Linkage map progeny	NA	NA	NA	NA	SRR29248736
**21126**	*L. vulgaris*	Unknown	Linkage map progeny	NA	NA	NA	NA	SRR29248735
**21128**	*L. vulgaris*	Unknown	Linkage map progeny	NA	NA	NA	NA	SRR29248734
**21130**	*L. vulgaris*	Unknown	Linkage map progeny	NA	NA	NA	NA	SRR29248733
**21132**	*L. vulgaris*	Unknown	Linkage map progeny	NA	NA	NA	NA	SRR29248732
**21134**	*L. vulgaris*	Unknown	Linkage map progeny	NA	NA	NA	NA	SRR29248731
**21136**	*L. vulgaris*	Unknown	Linkage map progeny	NA	NA	NA	NA	SRR29248730
**21137**	*L. vulgaris*	Unknown	Linkage map progeny	NA	NA	NA	NA	SRR29248729
**21138**	*L. vulgaris*	Unknown	Linkage map progeny	NA	NA	NA	NA	SRR29248727
**21139**	*L. vulgaris*	Unknown	Linkage map progeny	NA	NA	NA	NA	SRR29248726
**21141**	*L. vulgaris*	Unknown	Linkage map progeny	NA	NA	NA	NA	SRR29248725
**21145**	*L. vulgaris*	Unknown	Linkage map progeny	NA	NA	NA	NA	SRR29248724
**21147**	*L. vulgaris*	Unknown	Linkage map progeny	NA	NA	NA	NA	SRR29248723
**21148**	*L. vulgaris*	Unknown	Linkage map progeny	NA	NA	NA	NA	SRR29248722
**21151**	*L. vulgaris*	Unknown	Linkage map progeny	NA	NA	NA	NA	SRR29248721
**21152**	*L. vulgaris*	Unknown	Linkage map progeny	NA	NA	NA	NA	SRR29248720
**21153**	*L. vulgaris*	Unknown	Linkage map progeny	NA	NA	NA	NA	SRR29248719
**21154**	*L. vulgaris*	Unknown	Linkage map progeny	NA	NA	NA	NA	SRR29248706
**21156**	*L. vulgaris*	Unknown	Linkage map progeny	NA	NA	NA	NA	SRR29248704

**21157**	*L. vulgaris*	Unknown	Linkage map progeny	NA	NA	NA	NA	SRR29248703
**21158**	*L. vulgaris*	Unknown	Linkage map progeny	NA	NA	NA	NA	SRR29248702
**21159**	*L. vulgaris*	Unknown	Linkage map progeny	NA	NA	NA	NA	SRR29248701
**21160**	*L. vulgaris*	Unknown	Linkage map progeny	NA	NA	NA	NA	SRR29248700
**21161**	*L. vulgaris*	Unknown	Linkage map progeny	NA	NA	NA	NA	SRR29248699
**21162**	*L. vulgaris*	Unknown	Linkage map progeny	NA	NA	NA	NA	SRR29248698
**21163**	*L. vulgaris*	Unknown	Linkage map progeny	NA	NA	NA	NA	SRR29248697
**21164**	*L. vulgaris*	Unknown	Linkage map progeny	NA	NA	NA	NA	SRR29248696
**21169**	*L. vulgaris*	Unknown	Linkage map progeny	NA	NA	NA	NA	SRR29248695
**21170**	*L. vulgaris*	Unknown	Linkage map progeny	NA	NA	NA	NA	SRR29248692
**21172**	*L. vulgaris*	Unknown	Linkage map progeny	NA	NA	NA	NA	SRR29248691
**21173**	*L. vulgaris*	Unknown	Linkage map progeny	NA	NA	NA	NA	SRR29248690
**21174**	*L. vulgaris*	Unknown	Linkage map progeny	NA	NA	NA	NA	SRR29248689
**21175**	*L. vulgaris*	Unknown	Linkage map progeny	NA	NA	NA	NA	SRR29248688
**21176**	*L. vulgaris*	Unknown	Linkage map progeny	NA	NA	NA	NA	SRR29248687
**21177**	*L. vulgaris*	Unknown	Linkage map progeny	NA	NA	NA	NA	SRR29248686
**21178**	*L. vulgaris*	Unknown	Linkage map progeny	NA	NA	NA	NA	SRR29248685
